# OCT-SelfNet: a self-supervised framework with multi-source datasets for generalized retinal disease detection

**DOI:** 10.3389/fdata.2025.1609124

**Published:** 2025-07-29

**Authors:** Fatema-E Jannat, Sina Gholami, Minhaj Nur Alam, Hamed Tabkhi

**Affiliations:** Department of Electrical and Computer Engineering, University of North Carolina at Charlotte, Charlotte, NC, United States

**Keywords:** self-supervised, transformer, deep learning, SwinV2, autoencoder, OCT, classification, transfer learning

## Abstract

**Introduction:**

In the medical AI field, there is a significant gap between advances in AI technology and the challenge of applying locally trained models to diverse patient populations. This is mainly due to the limited availability of labeled medical image data, driven by privacy concerns. To address this, we have developed a self-supervised machine learning framework for detecting eye diseases from optical coherence tomography (OCT) images, aiming to achieve generalized learning while minimizing the need for large labeled datasets.

**Methods:**

Our framework, OCT-SelfNet, effectively addresses the challenge of data scarcity by integrating diverse datasets from multiple sources, ensuring a comprehensive representation of eye diseases. By employing a robust two-phase training strategy self-supervised pre-training with unlabeled data followed by a supervised training stage, we utilized the power of a masked autoencoder built on the SwinV2 backbone.

**Results:**

Extensive experiments were conducted across three datasets with varying encoder backbones, assessing scenarios including the absence of self-supervised pre-training, the absence of data fusion, low data availability, and unseen data to evaluate the efficacy of our methodology. OCT-SelfNet outperformed the baseline model (ResNet-50, ViT) in most cases. Additionally, when tested for cross-dataset generalization, OCT-SelfNet surpassed the performance of the baseline model, further demonstrating its strong generalization ability. An ablation study revealed significant improvements attributable to self-supervised pre-training and data fusion methodologies.

**Discussion:**

Our findings suggest that the OCT-SelfNet framework is highly promising for real-world clinical deployment in detecting eye diseases from OCT images. This demonstrates the effectiveness of our two-phase training approach and the use of a masked autoencoder based on the SwinV2 backbone. Our work bridges the gap between basic research and clinical application, which significantly enhances the framework's domain adaptation and generalization capabilities in detecting eye diseases.

## 1 Introduction

A significant gap exists between the progress made in AI technology and its applicability in real-world medical scenarios. The problem of applicability arises primarily due to the scarcity of labeled data in the medical domain, largely driven by privacy concerns. This limitation restricts the deployment of scalable medical AI solutions in diverse patient populations. Solving this issue is crucial for advancing AI-driven healthcare and ensuring that models can generalize to diverse clinical settings. Our work addressed these challenges by developing a machine learning (ML) tool to detect eye diseases using optical coherence tomography (OCT) images, which is vital for effective eye care management. In addition, we created a model that learns generalized features from diverse, unlabeled data, enhancing its applicability and reliability in real-world medical scenarios. Our contributions focus on overcoming data scarcity and ensuring the wide applicability of AI models in healthcare.

Age-related macular degeneration (AMD) is one of the leading causes of irreversible blindness and vision impairment (VI) globally. VI affects nearly 2.2 billion people globally, among which almost 1 billion cases could be prevented with early diagnosis and intervention (World Health Organization, 2023). Therefore, it is essential to identify individuals at risk of disease onset or progression from early to more advanced stages since timely intervention can prevent or slow progression, thus preventing irreversible VI (Scott and Bressler, 2013). Individuals with a high risk for VI would benefit from more frequent ophthalmic examinations, monitoring, and treatment (Yi et al., 2009).

In real-world clinical settings, the effectiveness of deep learning networks is often limited by the use of homogeneous training datasets. To enhance their performance, it is essential to use diverse datasets from multiple institutions with varying demographics, OCT devices, and protocols. This diversity will improve the models' adaptability and scalability in clinical workflows. Moreover, it will enable the model to be trained on a larger sample size, helping to mitigate the issue of data scarcity and further enhancing its generalization ability.

Self-supervised learning (SSL) is a new approach to computer vision, and advancements have been made in Natural Language Processing (NLP), specifically with the development of BERT (Devlin et al., 2018). SSL focuses on deriving meaningful information from unlabeled data. The masked autoencoder (MAE) (He et al., 2022) focuses on reconstructing masked portions of the input data. This approach allowed the model to learn robust feature representations by understanding the underlying structure of the visual data.

Our proposed work has brought and evaluated recent advances in large pre-trained transformers to enhance the detection and diagnosis for automated ophthalmic diagnosis. We have developed a large-scale, self-supervised model with random masking inspired by masked autoencoder (He et al., 2022) with a transformer architecture, SwinV2 (Liu et al., 2022) backbone, explicitly for classifying AMD using OCT images. Our approach builds on the current implementation of Masked Autoencoders (MAE) for OCT data by introducing two key differences that set it apart. First, while conventional MAEs typically rely on standard ViT architectures, we integrate the more advanced SwinV2 architecture, which offers superior feature extraction through its hierarchical attention mechanism, capturing both fine-grained and global structures in OCT images more effectively. Second, we enhance the pre-training phase of our OCT-SelfNet framework by incorporating a Data Fusion methodology, allowing our model to learn from multiple diverse datasets simultaneously. This contrasts with the standard MAE, where models are typically trained on a single dataset, potentially limiting their generalizability. By using data fusion, our model not only learns more diverse representations but is also better trained to generalize across different clinical datasets and imaging conditions, making it more acceptable for real-world applications. We focus on the binary classification of distinguishing normal cases from those with AMD.

By leveraging self-supervised learning (SSL), this model aims to reduce the need for experts to make extensive manual annotations, easing the workload and facilitating broader clinical AI applications from retinal imaging data. Our model can learn versatile and generalizable features from unlabeled retinal OCT datasets, which is crucial for creating AI systems that require fewer labeled examples to adapt to various diagnostic tasks.

Our framework follows a two-phase approach designed to optimize model performance by leveraging both self-supervised and supervised learning techniques. In the first phase, a SwinV2-based masked autoencoder undergoes self-supervised training using a combination of three different OCT datasets without any labels. This phase allows the model to learn generalized, low-level features by reconstructing masked portions of the input images, capturing valuable information across various datasets. By training without labels, the model can identify robust patterns shared across different clinical contexts, enhancing its ability to learn rich representations that are not biased by specific dataset annotations. In the second phase, the model undergoes a training in a supervised manner, focusing on each dataset. This phase enables the model to learn more dataset-specific features, training its weights to better align with the particular dataset it is working with. To assess the performance of different transformer models with our method, we also evaluated our results using Vision Transformer (ViT) (Dosovitskiy et al., 2020) and Swin Transformer-based masked autoencoders (Liu et al., 2021). ViT computes self-attention across the entire image, which requires quadratic time complexity *O*(*N*^2^) with respect to the number of patches (*N*). This is computationally expensive, especially for high-resolution images. Due to this global attention, ViT becomes inefficient for large images. In Swin, the shifted window attention mechanism is introduced, where self-attention is computed within local, non-overlapping windows, which reduces the computational complexity to *O*(*N*) per window. However, it allows for global context learning by shifting the windows across layers. SwinV2 introduces additional optimizations to reduce computational overhead further. It improves scalability, allowing the model to handle even larger images more efficiently without sacrificing performance. This comparison allowed us to observe how each transformer model adapted to our approach.

Studies demonstrate the adaptability of the ResNet50 model in handling complex tasks like AMD detection and diabetic retinopathy classification using OCT images, showcasing the effectiveness of this architecture in medical imaging analysis (Alam et al., 2020; Leingang et al., 2023; Sotoudeh-Paima et al., 2022; Xu et al., 2023). We used it as the reference model to compare with the result from our proposed methodology. In addition to using the ResNet50 model, we also employed the Vit-base model as another baseline model to conduct a comprehensive analysis. Multiple OCT datasets (Kermany et al., 2018; Srinivasan et al., 2014; Li et al., 2020), named DS1, DS2, and DS3, respectively, were combined to train an improved DL model to increase the training data's diversity and allow our model to learn a broader range of patterns and relationships. This diversity will improve the model's generalization to new, unseen data, and it will be beneficial for the cases where the larger dataset is unavailable.

**Figure 1:**
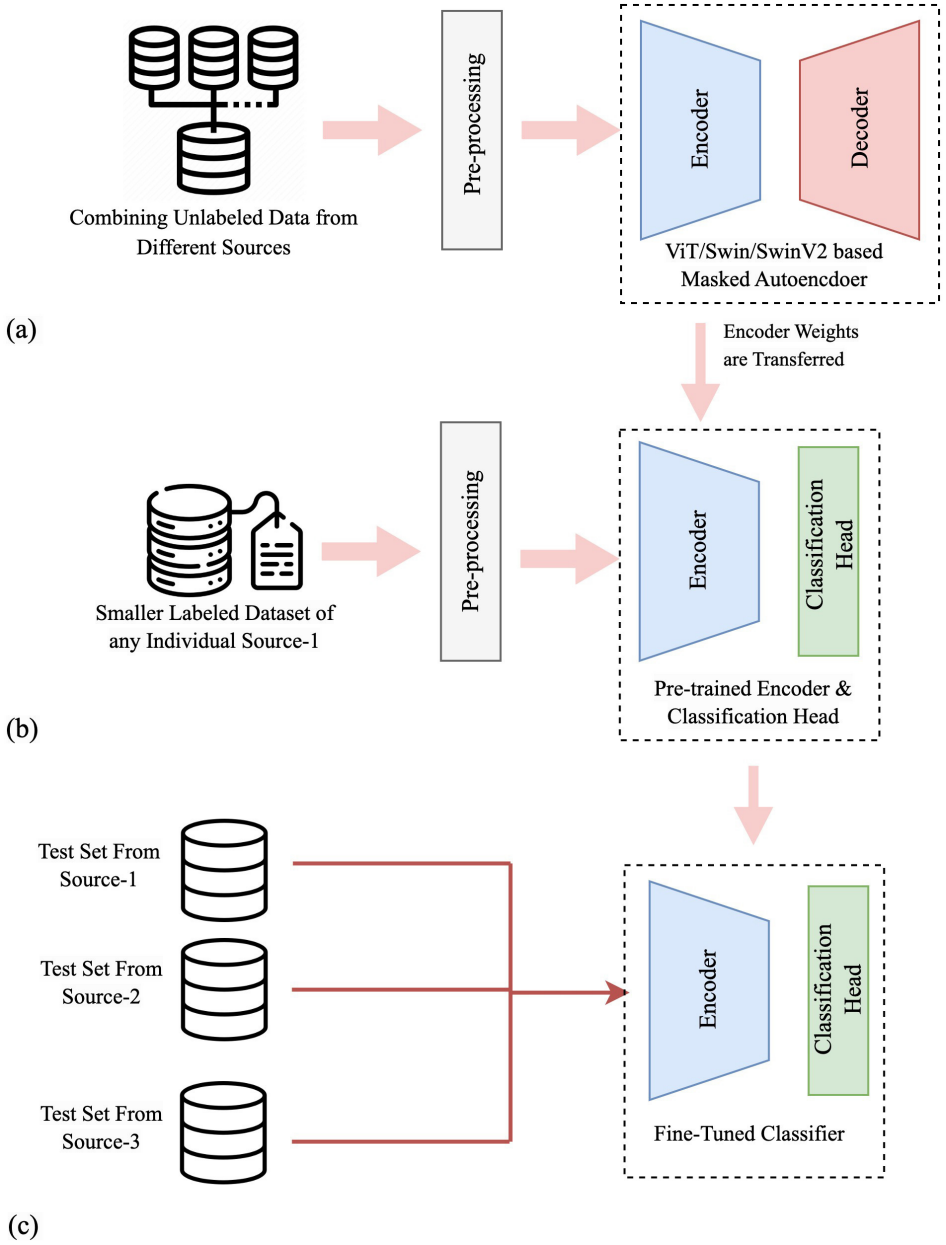
A graphical depiction of our methodology, with procedural steps. **(a)** Self-supervised pre-training using OCT images from multiple sources. **(b)** Supervised training on an individual dataset. **(c)** Performance and generalization evaluation on test sets from all sources.

A graphical depiction of our methodology, with procedural steps, is shown in Figure 1. Figure 1a illustrates self-supervised pre-training using OCT images from multiple sources. OCT images from training sets of three sources are combined without labels to create a fused dataset for the SSL pre-training. A masked autoencoder with a transformer-based network consisting of an encoder and a decoder parts is used in this phase. Figure 1b shows training on an individual dataset, where the decoder part is replaced with a classification head, and pre-trained weights are transferred to the encoder. Figure 1c depicts the performance and generalization evaluation on test sets from all sources using the trained classifier model. Specifically, if the classifier is trained using DS1, it is subsequently evaluated on all three test sets. This evaluation aims to observe how well the model performs on test sets other than the one it was trained on, thereby assessing its generalization capability as a supervised classifier and cross-dataset generalizer across diverse datasets.

The main contributions of this paper are summarized as follows:

- This paper implemented a two-phase approach: (1) a SwinV2-based masked autoencoder in the pre-training phase to deeply understand image structures and relationships between different regions, and (2) a supervised training stage classifier that is for classifying specific age-related macular degeneration (AMD) cases from normal cases using OCT images.
- This paper incorporates a Data Fusion methodology into its framework to enhance the capability of the model by combining information from multiple sources, which allows for more robust and comprehensive analysis, improving the model's ability to generalize and make accurate predictions across small datasets and clinical scenarios.
- Through a comprehensive evaluation and ablation study, this paper demonstrates that the proposed approach shows much higher robustness and better generalization even when evaluated on different test sets without requiring additional training. This is promising for effective implementation in practical clinical environments.
- This paper also evaluates OCT-SelfNet as a cross-dataset generalizer, where it exceeds the baseline model's performance, further demonstrating its strong generalization ability in handling unseen data.

## 2 Related works

Machine Learning (ML) and Deep Learning (DL) techniques have shown promise in automating ophthalmic diagnosis. Significant research has been done on longitudinal OCT retinal layer segmentation through the use of an LSTM-based U-Net architecture (He et al., 2023). Additionally, the work by Mukherjee et al. (2022a) and Mukherjee et al. (2022b) has leveraged advanced techniques such as 3D U-Net and 3D convolutional autoencoders, for retinal layer segmentation in OCT images.

Implementing a self-supervised framework using transformer-based encoder networks with multi-source datasets for generalized retinal disease detection signifies a notable progression in medical image analysis. This approach leverages the power of transformer models (Vaswani et al., 2017), originally introduced in natural language processing (NLP) and adapted for computer vision tasks such as medical imaging by opening opportunities for scalable and generalized models and sparked significant interest among researchers in computer vision. Inspired by this, the vision transformer (ViT) (Dosovitskiy et al., 2020) has been developed. It has been extensively studied in medical imaging (Alshammari et al., 2022; Ayana et al., 2023; Kihara et al., 2022; Okolo et al., 2022; Wang et al., 2022). However, employing a ViT architecture requires significant computational resources, posing a challenge for communities with limited computational infrastructure. Moreover, the necessity of substantial datasets for efficient training presents challenges in scenarios with limited data availability, particularly in the medical field. Despite these challenges, researchers are actively working on solutions to address these issues through continuous advancements in hardware, developing efficient techniques and algorithms.

In contrast to previous works (Awais et al., 2017; Leandro et al., 2023; Lee et al., 2017; Lu et al., 2018; Tsuji et al., 2020), which study one dataset in isolation, our study is distinct and intrinsically more challenging as we investigate the intricacies of domain adaptation by simultaneously considering multiple datasets, training our model on one dataset, and assessing its performance on other datasets to understand domain generalization and adaptation dynamics.

The evolution of SSL in computer vision progressed significantly with the introduction of BEiT (Bao et al., 2021), which incorporated BERT-like pre-training methods into image processing. BEiT's approach to predicting masked portions of images illustrated a significant advancement in self-supervised learning paradigms, bridging the gap between language and vision modalities. Other works underlined (Fang et al., 2022; Jing and Tian, 2020; Qiu and Sun, 2019) the growing significance of SSL in ophthalmology-focused deep learning research. They demonstrate how SSL can be leveraged to overcome challenges such as the scarcity of labeled data and the need for patient-specific diagnostic tools. In the field of Cervical OCT image classification, the work by Wang et al. (2024) has used this SSL combining contrastive-MIM framework and Swin transformer architecture to use unlabeled cervical OCT images. MedFLIP, developed by Li et al. (2024), has integrated a Masked Auto Encoder with a text encoder to facilitate mutual learning between text and image modalities in medical image analysis. For the analysis of smaller CT scan datasets, Wolf et al. (2023) focused on self-supervised pre-training using contrastive and masked autoencoders to leverage a large, unannotated CT dataset during the pre-training phase. Zhou et al. (2023) also used this SSL framework to train on medical images and subsequently employed the pre-trained model for downstream tasks such as chest X-ray disease classification, abdominal CT multi-organ segmentation, and MRI brain tumor segmentation.

Automated ophthalmic diagnosis benefits significantly from the application of machine learning techniques, as indicated by research (Alam et al., 2020; Friberg et al., 2011; Schmidt-Erfurth et al., 2018; Wang et al., 2016). However, relying on similar datasets during training often hinders their real-world effectiveness, resulting in challenges when deployed in clinical settings. To optimize their effectiveness in clinical workflow, they require access to diverse datasets from multiple institutions with varying demographics, OCT image-capturing devices, or protocols to improve their adaptability, versatility, and scalability. Our framework employs the data fusion methodology, and in an ablation study, the noticeable performance decline without data fusion highlights the necessity of leveraging diverse datasets. This aspect is particularly advantageous in enhancing the model's generalization capabilities, making it adept at handling unseen data and variations in image settings commonly encountered in clinical scenarios.

## 3 Methodology

In this work, we have leveraged pre-trained weights from an SSL MAE network (He et al., 2022) with SwinV2 (Liu et al., 2022) as a backbone, and combined multiple datasets, demonstrating a two-stage approach concerning the classification of Normal vs. AMD from OCT images by comparing their performance against the baseline model. Our proposed framework comprises four integral stages.

1. Data fusion: a comprehensive approach by integrating datasets from three different sources has been adopted to enhance the data diversity.
2. Self-supervised pre-training: in this stage, self-supervised pre-training was conducted on unlabeled OCT images using transformer-based MAE to acquire visual representations. After completing this training, the learned weights were transferred to a classifier model.
3. Supervised training: subsequently, supervised training was performed on labeled OCT datasets using the transferred weights to enhance the model's classification capabilities.
4. Baseline training: we have used ResNet50 (He et al., 2015), and ViT-base (without SSL) (Dosovitskiy et al., 2020) classifier as the baseline model to compare the performance of the proposed models.

### 3.1 Data fusion

In this step, we used three datasets containing Optical Coherence Tomography (OCT) images that illustrate various retinal diseases. For this study, we focused on binary classification and therefore only retained OCT images of NORMAL and AMD cases, and the rest of the categories of OCT images were removed. Then we split each of those datasets into training, validation, and test sets. All training data from the three datasets was combined and shuffled into a single training set, and all validation sets were similarly combined. Test sets were excluded from the data merging process to maintain the model's complete separation from the test sets, which will later be used for evaluation purposes in the later stage. These combined training and validation sets are used in the self-supervised pre-training stage.

The motivation behind this data combination is to increase the diversity of the training data so that the model can learn larger representations in the self-supervised pre-training stage, and it will help the model to generalize better for the case of an unseen dataset. During the supervised stage, the trained model will be trained using three separate datasets. Each training session will focus on an individual training set, with evaluations conducted on the test set of that dataset as well as on the test sets from the other two datasets. This approach will help assess the performance of the classifier.

### 3.2 Self-supervised pre-training

SSL is a technique that enables a model to train itself from unlabeled data by understanding the structure or representation of the data. An example of an SSL technique is the MAE (He et al., 2022), which randomly masks some parts of the input data and trains the model to learn the representation of the given data to reconstruct the original input.

The MAE trained in this study was primarily composed of two components: an encoder and a decoder. The image, resized to (224 × 224) was fed into the encoder portion of the MAE, which then applied a patch operation (16 × 16 patches) to randomly mask a portion (70%) of the input image and finally processed it through a transformer encoder. We chose to use random masking as part of the self-supervised learning (SSL) approach because it is a simple yet effective strategy for encouraging the model to learn robust feature representations without requiring labeled data. Random masking allows the model to focus on different regions of the image during training. During the experimentation phase, we conducted a series of trials to explore different masking ratios, including 20%, 50%, 70%, and 80%. Based on our results, we observed that masking 70% of the image produced the best performance in terms of model accuracy and generalization. Therefore, we settled on the 70% masking ratio as it provided the most balanced trade-off between model performance and computational efficiency. This 70% masking ratio was found to be optimal as it strikes a good balance between covering a significant portion of the image for effective training and preserving enough visible content for the model to focus on important features.

In the encoder, we used three distinct networks–ViT (Dosovitskiy et al., 2020), Swin (Liu et al., 2021), and SwinV2 (Liu et al., 2022) as a backbone to conduct a comprehensive study on their performance.

1. ViT-based MAE: in ViT-based MAE, the encoder comprised an embedding dimension of 1,024 and four attention heads, repeated for six layers. The encoder's final output was the set of features representing a higher-level abstraction of the original input image. The features were taken as input and processed by the decoder through the transformer layers. The transformer had an embedding dimension of 1,024 and four attention heads, which were repeated for four layers. After passing through a linear layer to get the patches, masking was applied, and finally, the image was reconstructed.
2. Swin-based MAE: the Swin transformer-based MAE uses an encoder built with a Swin transformer backbone with an embedding size of 96. The number of layers in each stage of the Swin transformer architecture is (2, 2, 18, 2), which indicates the number of layers at each stage. The model uses shifted window attention mechanisms in each stage to focus on local information within 4 × 4 patches. This gradually builds a global understanding by connecting windows with shifts. The attention heads are set to (6, 12, 24, 48), which doubles with each stage. This enables the model to attend to more intricate details, indicating that different layers of the encoder employ varying attention heads, capturing hierarchical features in the input image.

The decoder had an embedding size of 768, which allowed for a more expressive representation in the decoding process. The decoder network had a similar number of attention heads and layers as the encoder. It involved a patch-expanding mechanism and Swin transformer layers, which were configured to restore the spatial dimensions of the encoded features. The layer-wise design was built to gradually reconstruct the original image dimensions, ensuring that the decoder could effectively decode the encoded representation obtained by the encoder.
3. SwinV2-based MAE: we used a SwinV2-based MAE for this task, taking advantage of the SwinV2 network's superior performance. While we kept the Swin-based decoder, we switched the encoder for SwinV2 to address issues with training stability, high-resolution processing, and data efficiency. SwinV2's improved performance over Swin is perfect for our need for both detail and efficiency. The encoder configuration was set using an embedding dimension of 96, depths of (2, 2, 6, 2), and attention heads of (3, 6, 12, 24). This tailored approach allowed the SwinV2-based MAEs to excel at extracting intricate details.

### 3.3 Supervised training

Following the self-supervised pre-training with the combined training samples, a transfer learning approach is applied in this supervised stage. For the classifier architecture, a classification head was added to the model in place of the decoder to use MAE as a classifier. The classification head took the features from the encoder part and ran through a linear layer to produce class logits. These class logits were then used for classification tasks. The linear layer comprised three successive dense layers, each accompanied by Rectified Linear Unit (ReLU) activation functions. This linear layer learned weights during training. The softmax function was then used to transform the class logits into class probabilities, enabling the model to predict the respective classes. While training, we applied the feature extraction methodology, where the pre-trained encoder weights were kept frozen, and only the classification head layers were trained. This choice was intentional, as it is more suited to real-life implementations, especially when dealing with limited data. Freezing the encoder allows us to leverage the robust capabilities of pre-trained models, even on small datasets, and the model can still perform effectively. Moreover, this approach significantly reduces training time, which is crucial in real-world applications where computational resources and time are often constrained.

**Figure 2:**
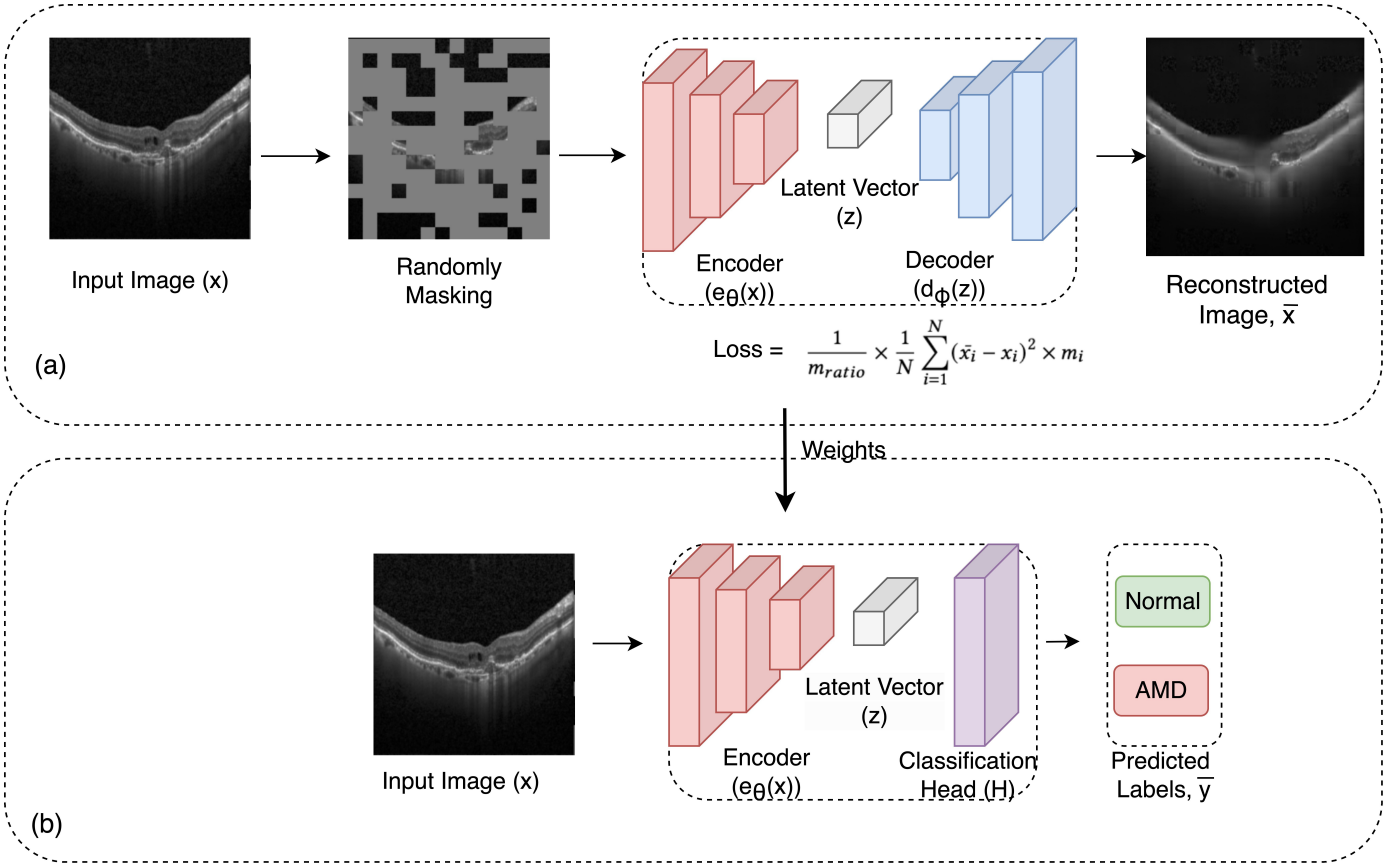
Overview of the framework: in the initial pre-training phase **(a)**, the framework uses masked image autoencoder as a self-supervised task to learn representations from unlabeled images. In this process, a random subset of image patches is masked and fed into the auto-encoder. Then in the subsequent supervised training stage **(b)**, the pre-trained encoder from the first phase is employed along with a linear classifier for the classification task. The learned weights from the pre-training phase are transferred to this phase.

The approach involved training one dataset and then evaluating the model's performance on the test set and two other test sets from other datasets to assess its robustness. This cross-evaluation procedure was repeated for all three datasets, comprehensively analyzing the model's adaptability across diverse datasets. In Figure 2, the two-stage training process is illustrated.

Integrating self-supervised pre-training and supervised training in this methodology establishes a robust framework, OCT-SelfNet, for classifying retinal diseases in OCT images. Combining the MAE architecture and the subsequent classifier model aims to capitalize on the learned representations to improve the model's generalization to new, unseen data.

### 3.4 Baseline model

For the comparison, we used ResNet-50 as our baseline model. Unlike our proposed approach, ResNet-50 did not undergo a self-supervised pre-training stage. Instead, we used a version of ResNet-50 that was pre-trained on the ImageNet dataset. This allowed us to evaluate the performance differences between a model with conventional pre-training and our self-supervised pre-training methodology. This ResNet-50 architecture started with a 7 × 7 kernel convolution and a max pooling layer, followed by a series of convolutional layers with varying sizes and numbers of kernels. Repeated in specific patterns, these layers enhanced the network's ability to extract and process complex features from images. After 50 convolutional layers, the network concluded with average pooling and fully connected layers with two nodes using softmax activation for binary classification (Normal vs. AMD). To provide a more comprehensive evaluation, we extended our baseline comparison by incorporating the ViT-Base (Vision Transformer) model (without the SSL pre-training). The choice of ViT-Base is motivated by its strong performance in various computer vision tasks, leveraging transformer-based architectures that are fundamentally different from the convolutional operations in ResNet50. By including ViT-Base, and ResNet50, we aim to evaluate the performance of our model against two state-of-the-art architectures.

The complete algorithm for the two-phase training process is presented in pseudocode (Algorithm 1) for clearer understanding.

**Algorithm 1:**
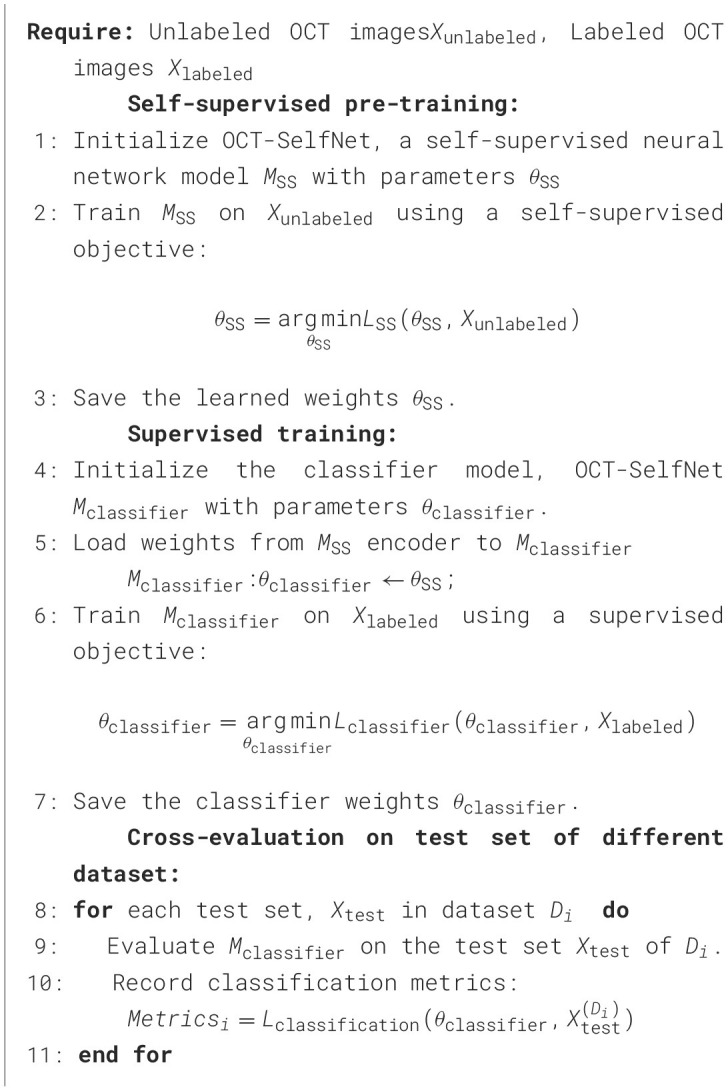
An algorithm for self-supervised pre-training and supervised training for OCT classification.

Table 1 details the network size and floating point operations per second (FLOPS) of the proposed and baseline models. It can be seen that the proposed OCT-SelfNet-SwinV2 classifier model has a slightly larger size than the baseline ResNet-50 classifier, but its FLOPS is very close to the ResNet-50. This demonstrates that while the proposed model introduces some additional parameters (34.27M) than ResNet-50 (23.5M), it still is much smaller than the ViT-base model (86M) and does not increase the computational load, making it comparable to the smaller ResNet-50 model in terms of efficiency.

**Table 1:** Network details.

**Mode**	**Model**	**Model size (M)**	**FLOPS (G)**
Pre-training	OCT-SelfNet-ViT	126.88	10.06
	OCT-SelfNet-Swinlarge	83.56	16.41
	OCT-SelfNet-SwinV2	33.79	5.62
Classifier	Resnet-50	23.5	4.1
	ViT-base	86	16.85
	OCT-SelfNet-ViT	60.52	10.47
	OCT-SelfNet-Swinlarge	84.01	8.55
	OCT-SelfNet-SwinV2	34.27	3.34

### 3.5 Loss function

We have employed a loss function for the pre-training phase that uses the mean squared error (MSE) between the predicted and original images and only considers the pixels where the mask is active. To determine the loss on the pixels where the mask is active, the mean square error (MSE) is multiplied by the mask. The percentage of the image that is masked is shown by the mask ratio. The loss is computed by dividing the mean square error (MSE) by the mask ratio because the mask is being used to focus only on particular regions of the image. This enables us to scale the loss appropriately and appropriately normalize the loss to the portion of the image that is masked. The loss function is provided in Equation 1,


(1) $$\begin{array}{l} \frac{1}{m_{ratio}}\times \frac{1}{N}\overset{N}{\underset{i=1}{\sum }}{\left(\bar{x_{i}}-x_{i}\right)}^{2}\times m_{i} \end{array}$$

Here $\bar{x_{i}}$ is the predicted image, *x*_*i*_ is the original input image, *m*_*i*_ is the mask, *m*_*ratio*_ is the mask ratio and N is the number of total sample.

## 4 Datasets

Our experiments were carried out on three distinct datasets, DS1, DS2, and DS3 to ensure a comprehensive evaluation of the proposed methodology for binary image classification (Normal vs. AMD). The use of those different datasets enables the training of models using varied distributions and provides richer comparisons of model performance across various test sets. In each of these datasets, there are multiple categories such as Normal, AMD, CNV, etc. Each OCT image is associated with a single disease category. For our study, we only considered the Normal and AMD categories from each dataset. Since our primary objective for this project was to develop a binary classifier for distinguishing NORMAL and AMD, we focused exclusively on these two classes of OCT images, removing other categories from both the training and evaluation processes.

### 4.1 DS1

DS1 encompasses a total of 109,559 OCT retinal (Spectralis OCT, Heidelberg Engineering, Germany) images which are classified into four categories: Normal, Choroidal Neovascularization (CNV), Diabetic Macular Edema (DME), and Drusen. We discovered identical images as stated before in this study (Gholami et al., 2023). So we followed their approach to clean the data and after that, we were left with 101,565 images. While Drusen are not an early sign of CNV, their presence in the retina can indicate a higher risk for developing advanced AMD, including the wet form which eventually involves the growth of abnormal blood vessels beneath the retina, a process known as CNV. Since Drusen is primarily associated with the early stage of AMD, we annotated Drusen images as AMD. Then we split this DS1 intro train, test, and validation set with 80%, 10%, 10% ratio.

### 4.2 DS2

The DS2 dataset includes retinal images from 45 subjects, consisting of 15 normal patients, 15 patients with dry AMD, and 15 patients with DME. We split this dataset into training, validation, and test sets, with the first 10 subjects allocated to training, the subsequent two subjects to validation, and the final three subjects to testing. All OCT volumes were obtained using Heidelberg Engineering Spectralis SD-OCT in protocols approved by the IRB (Srinivasan et al., 2014).

### 4.3 DS3

DS3 is a dataset comprising OCT images from 500 subjects. These images were captured using two different fields of view: 3- and 6-mm. A single 3-mm file contains 304 scans of an individual patient, while a 6-mm file contains 400 scans. Our focus was on the slice images of the fovea since they capture the most prominent features of the retina. We considered peripheral retinal sections to have limited significance in classification. All OCT images were captured using a spectral-domain OCT system with a center wavelength of 840 nm (RTVue-XR, Optovue, CA) (Li et al., 2020). This DS3 was divided into training, validation, and test sets using a stratified approach based on disease categories (AMD and Normal). Each disease category was grouped separately, and then the dataset was split into three subsets: 80% for training, 10% for validation, and 10% for testing, ensuring an equal distribution of samples across all disease categories. This stratified splitting approach helps maintain representative distributions of diseases in each subset, which is essential for effectively training and evaluating machine learning models.

During the self-supervised pre-training stage, the model was trained with training sets from those three datasets to keep the test set unseen from the model. Also, all classes from those three datasets have been used in this pre-training stage, allowing it to grasp the intricacies of representation learning comprehensively. This inclusive training approach enabled the network to capture a broad spectrum of features and patterns present in the diverse classes. However, in the supervised training stage, we focused on binary classification tasks concentrating solely on AMD and Normal categories. This two-phase training approach, from comprehensive pre-training to feature extraction, strategically guides the model's representation learning and optimizes its performance for the targeted binary classification objective.

**Figure 3:**
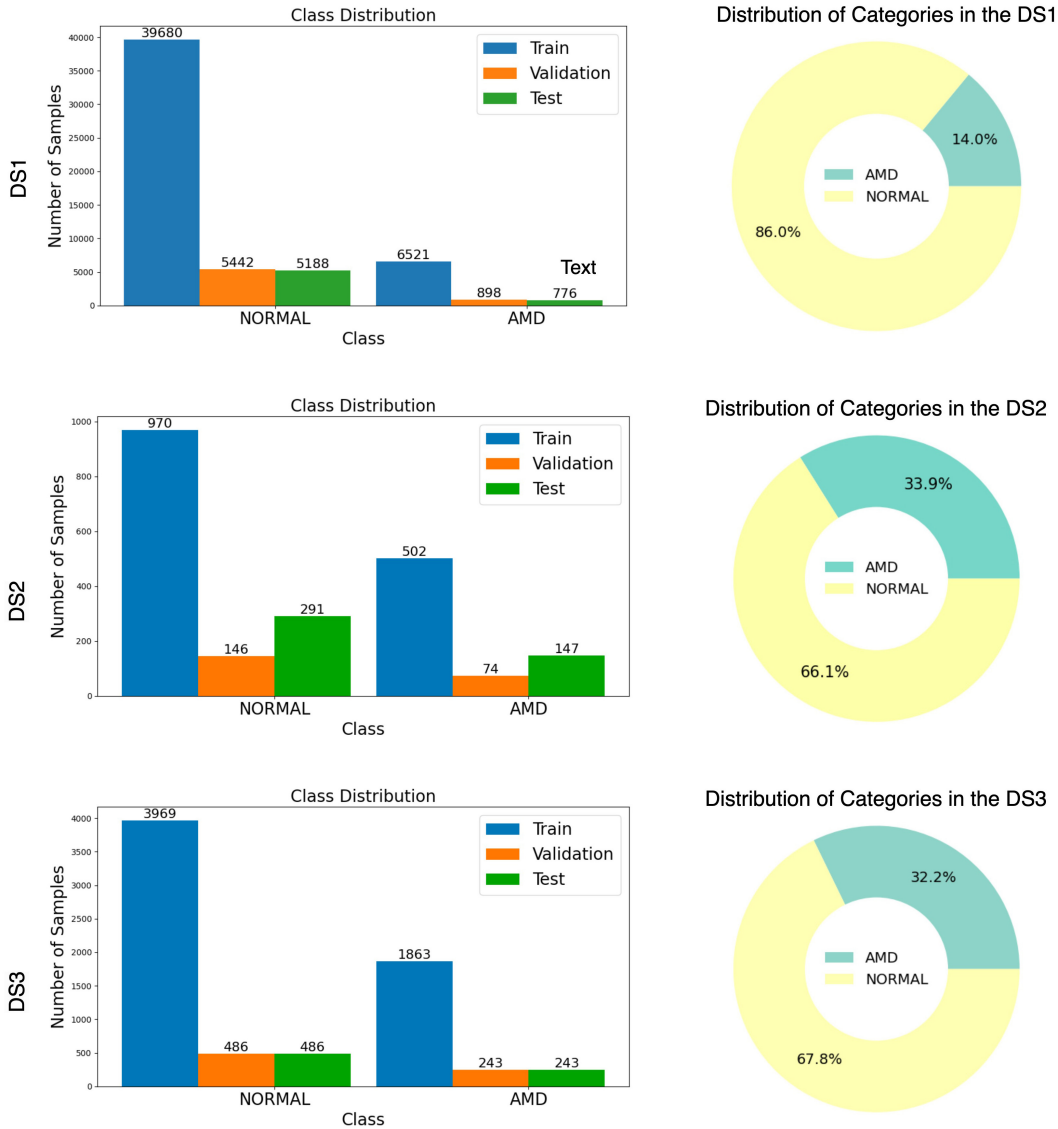
Illustration of Normal and AMD distribution from three datasets (DS1, DS2, and DS3) with bar chart and donut chart.

A general overview of these three datasets is provided in Figure 3, with the distribution of data across the training, validation, and test sets, along with the count of normal and AMD in each set, accompanied by a bar chart and donut chart.

## 5 Experiments

### 5.1 Implementation details

The learning rate is set to 1.5 × 10^−4^ and Adam optimizer with weight decay (Loshchilov and Hutter, 2017) of 0.05, using β_1_ and β_2_ values of 0.9 and 0.95, respectively. The input consists of a batch of 32 images, which were normalized. The NVIDIA Tesla V100 graphical processing unit (GPU) was used for the experiments. In the self-supervised pre-training stage, the model was run for 50 epochs, and the model with the minimum validation loss was saved for subsequent training. For the supervised training stage, the model was run for 100 epochs with early stopping criteria with a patience of 10, and the model with the maximum validation accuracy was saved for testing. All the baseline experiments were done and evaluated on an NVIDIA GeForce RTX 3060 Ti GPU using similar hyperparameters. During supervised training stage random rotation, horizontal flip, color jittering, Gaussian blurring, and elastic transform techniques were used for data augmentation.

All images were resized to 224 × 224 in training, and codes were implemented with CUDA 11.2, Pytorch 1.12.1, and Python 3.10.9.

### 5.2 Evaluation metrics

The distribution Figure 3 reveals that all three datasets are highly imbalanced, with the NORMAL cases significantly outnumbering the AMD cases. This imbalance makes accuracy an inadequate measure of the model's performance, as it could be misleadingly high simply due to the prevalence of NORMAL cases. Instead, the AUC-ROC score is a more appropriate metric, accounting for both true positive and false positive rates across various threshold settings. To provide a comprehensive evaluation, we also used the Area Under the Precision-Recall Curve (AUC-PR) and the F1-Score and Wilcoxon Signed-Ranking. These metrics offer further insights into the model's ability to handle imbalanced data by considering the precision and recall, which are critical for understanding the model's performance in distinguishing between the minority and majority classes.

1. Accuracy: accuracy measures how many correct predictions were made by the model; it is calculated by dividing the total number of correct predictions by the total number of predictions. The formula for accuracy is given by Equation 2.

(2) $$\begin{array}{l} Accuracy=\frac{TP+TN}{TP+TN+FP+FN} \end{array}$$

Here, TP = true positives, TN = true negatives, FP = false positives, and FN = false negatives.
2. AUC-ROC: the area under the receiver operating characteristic curve (AUC-ROC) is a metric used to evaluate a binary classifier. The ROC curve plots the true positive rate against the false positive rate for different threshold values. The AUC-ROC is the area under the ROC curve, which gives a single value that summarizes the overall performance of the model across various threshold settings. Higher AUC-ROC indicates better discrimination of positive and negative classes.
3. AUC-PR: similar to AUC-ROC, the Area Under the Precision-Recall curve (AUC-PR) is a performance metric used to evaluate a binary classifier. PR curve focuses on precision and recall by plotting precision against recall for different threshold values. The AUC-PR is the area under the Precision-Recall curve, which provides a single value to denote the model's overall performance across various threshold settings. The higher the score, the better the performance.
4. F1-score: F1-score is another performance evaluation metric that takes both precision and recall into consideration which makes this metric very useful in the case of data imbalance. The F1-score is calculated using the Equation 3.

(3) $$\begin{array}{l} F1Score=\frac{2*Precision*Recall}{Precision+Recall} \end{array}$$

The value of F1-score ranges from 0 to 1, where a higher value indicates better performance.
5. Wilcoxon Signed-Rank test: a statistical test has been done to assess whether the observed performance differences (e.g., between our proposed model, OCT-SelfNet, and the baseline models) are statistically significant or merely due to random variation. To evaluate this, the *p*-value for AUC-ROC scores between OCT-SelfNet and the baseline models in each of our experiments is calculated. Given that AUC-ROC scores are not guaranteed to follow a normal distribution and the data is paired, the Wilcoxon Signed-Rank Test is used. A *p*-value < 0.05 indicates a statistically significant difference, meaning the observed improvement or difference is unlikely to be due to chance and vice versa.

### 5.3 Self-supervised pre-training result

**Figure 4:**
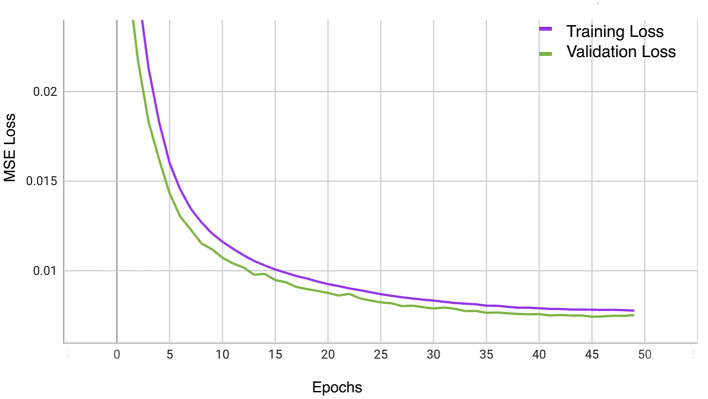
The training and validation MSE Loss curves of OCT-SelfNet with SwinV2 backbone.

**Figure 5:**
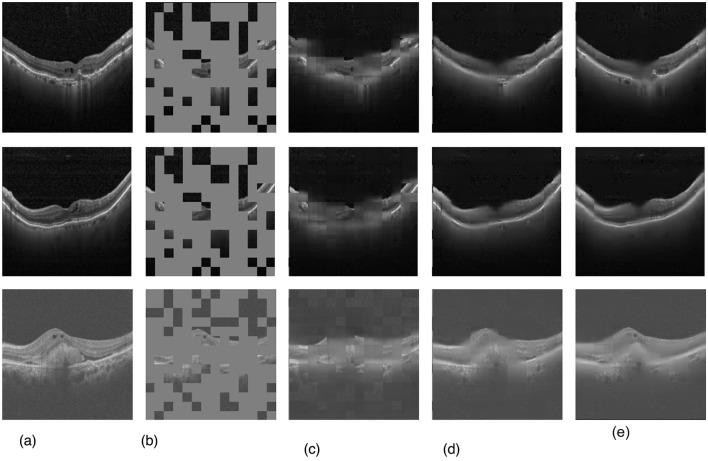
Qualitative visualizations of the performance of SwinV2-based self-supervised MAE pre-training. **(a)** Ground truth image, **(b)** input image with masks, **(c)** after epoch-2, **(d)** after epoch-20, and **(e)** final reconstructed image after epoch-50.

Our evaluation explored the efficacy of three transformer-based networks–ViT, Swin, and SwinV2–by conducting pre-training for 50 epochs. Specifically, the SwinV2-based MAE exhibited notable proficiency, achieving a mean squared error (MSE) loss of 0.007 after 50 training epochs, as depicted in Figure 4. Figure 5 illustrates a visualization of the OCT image reconstruction from the SwinV2-based MAE for different epochs. These qualitative visualizations demonstrate the performance of the Swinv2-based self-supervised Masked Autoencoder (MAE) during pre-training. The visualizations consist of a sequence of images arranged from left to right: starting with the ground truth image, followed by the input image where random regions are masked, and reconstructed images showing predicted patches at different epochs (epoch-2, epoch-20, and epoch-50). This series illustrates the model's progressive learning process in reconstructing the masked regions over time. Initially, the reconstructed images show rudimentary patch predictions at epoch-2, gradually improving in accuracy and detail by epoch-20. After epoch 50, the model achieves more refined reconstructions, though the predicted patches in the reconstructed image may not be entirely clear. However, our project's primary objective was not to generate flawless reconstructions but to capture intricate image structures and patterns. In subsequent tasks, we used these pre-trained weights, leveraging their learned representations rather than initializing the classifier network randomly.

### 5.4 Supervised training result

#### 5.4.1 Performance comparison with different encoder networks

To observe how different transformer model adapts to our approach, in this experimentation, we employed three diverse transformer-based MAE networks during the pre-training phase. In the next stage, self-supervised training, we maintained the same encoder and leveraged transfer learning to transfer the learned weights. Additionally, a classifier network was integrated for the downstream classification tasks. Each supervised network underwent training for every dataset, and evaluations were conducted across all three test sets to assess the network's performance on previously unseen test data. The performance was then compared with the baseline model ResNet-50, which underwent training on each dataset and subsequent evaluation on all three test sets. Data augmentation techniques were applied throughout this experimental process. Performance metrics, including Accuracy, AUC-ROC, AUC-PR, and F1-Score, were employed to measure the effectiveness of the different encoders.

Analysis of Table 2 shows that, in general classification tasks, the performance of our self-supervised training approach is comparable to that of the baseline models. However, as demonstrated in Table 3, our method exhibits superior performance in cross-dataset generalization analysis. In this evaluation, the model was trained on DS1, and its classification performance was assessed on DS2 and DS3. Similar evaluations were conducted for DS2 and DS3 as well. The results in the table demonstrate that our method outperforms the baseline models in most cases, highlighting the superior generalization capability of our approach compared to traditional models.

**Table 2:** Comparison of our work with the baseline methods (ResNet-50, and ViT) with three datasets.

**Dataset**	**Classifier name**	**Accuracy**	**AUC-ROC**	**AUC-PR**	**F1-score**
DS1 (Test-1)	ResNet50	**0.99**	**0.98**	**0.97**	**0.97**
	ViT	0.96	0.97	0.91	0.83
	OCT-SelfNet-ViT	0.94	0.94	0.84	0.75
	OCT-SelfNet-Swinlarge	0.96	0.96	0.90	0.83
	OCT-SelfNet-SwinV2	0.96	0.96	0.89	0.84
DS2 (Test-2)	ResNet50	0.87	0.80	0.86	0.76
	ViT	0.87	0.93	0.90	0.82
	OCT-SelfNet-ViT	0.90	0.97	0.94	0.87
	OCT-SelfNet-Swinlarge	0.92	0.99	0.96	0.90
	OCT-SelfNet-SwinV2	**0.99**	**0.99**	**0.99**	**0.98**
DS3 (Test-3)	ResNet50	0.95	0.96	0.94	0.94
	ViT	**0.98**	**0.99**	**0.99**	**0.98**
	OCT-SelfNet-ViT	0.94	0.98	0.97	0.91
	OCT-SelfNet-Swinlarge	0.97	**0.99**	**0.99**	0.96
	OCT-SelfNet-SwinV2	**0.98**	0.98	0.97	0.97

The evaluation focuses on binary classification accuracy, AUC-ROC, AUC-PR, and F1-score. Scores shown in bold represent the highest performance.

**Table 3:** Comparison of classifiers for DS1, DS2, and DS3 datasets for cross dataset generalization analysis.

**Train set**	**Classifier name**	**Accuracy**		**AUC-ROC**		**AUC-PR**		**F1-score**	
		**Test-2**	**Test-3**	**Test-2**	**Test-3**	**Test-2**	**Test-3**	**Test-2**	**Test-3**
DS1	ResNet50	0.98	0.70	**0.99**	0.56	0.97	0.70	0.98	0.20
	ViT	0.98	**0.97**	**0.99**	**0.99**	**0.99**	**0.99**	0.98	**0.95**
	OCT-SelfNet-ViT	0.95	0.78	**0.99**	0.87	0.97	0.76	0.96	0.51
	OCT-SelfNet-Swinlarge	0.97	0.77	**0.99**	0.87	**0.99**	0.76	0.96	0.51
	OCT-SelfNet-SwinV2	**0.99**	0.88	**0.99**	0.93	**0.99**	0.86	**0.99**	0.80
		**Test-1**	**Test-3**	**Test-1**	**Test-3**	**Test-1**	**Test-3**	**Test-1**	**Test-3**
DS2	ResNet50	**0.77**	0.42	0.59	0.54	0.33	0.64	0.28	0.51
	ViT	0.64	0.47	0.66	0.71	0.24	0.55	0.30	0.53
	OCT-SelfNet-ViT	0.71	0.54	0.74	0.85	0.30	0.67	0.35	**0.67**
	OCT-SelfNet-Swinlarge	0.65	**0.55**	0.75	0.76	0.26	0.58	0.37	0.57
	OCT-SelfNet-SwinV2	0.72	**0.55**	**0.79**	**0.86**	**0.42**	**0.73**	**0.39**	0.59
		**Test-1**	**Test-2**	**Test-1**	**Test-2**	**Test-1**	**Test-2**	**Test-1**	**Test-2**
DS3	ResNet50	0.86	0.87	0.72	0.87	**0.53**	0.84	**0.49**	0.82
	ViT	0.68	0.93	0.74	**0.96**	0.36	**0.96**	0.34	0.88
	OCT-SelfNet-ViT	0.88	0.85	**0.77**	0.90	0.46	0.88	0.39	0.71
	OCT-SelfNet-Swinlarge	0.86	**0.94**	0.75	0.95	0.45	0.95	0.44	**0.91**
	OCT-SelfNet-SwinV2	**0.88**	0.83	0.75	0.93	0.44	0.84	0.46	0.72

Results of our work (OCT-SelfNet-ViT) are compared with baseline methods (ResNet-50, ViT) on binary classification. Scores shown in bold represent the highest performance.

**Figure 6:**
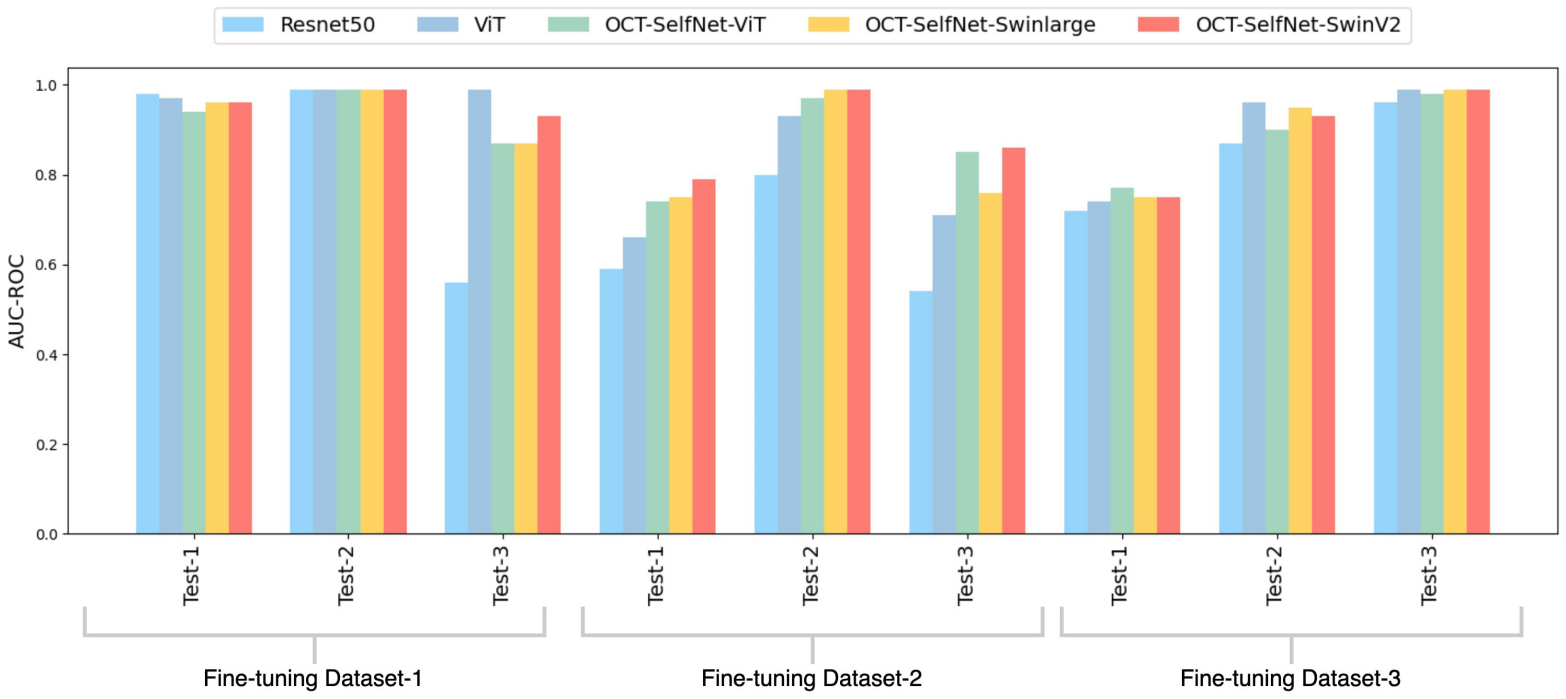
Comparison of AUC-ROC scores for different classifiers across three datasets and test sets. Each group represents AUC-ROC scores for classifiers within a specific dataset and test set combination, highlighting performance variations. Comparison of AUC-ROC scores for different classifiers across three datasets and test sets. Each group represents AUC-ROC scores for classifiers within a specific dataset and test set combination, highlighting performance variations.

The bar chart depicted in Figure 6 presents a comparison of AUC-ROC scores among various classifiers across three datasets and test sets. The grouped bars allow for a direct comparison of each classifier's performance, showcasing OCT-SelfNet's superior performance over the baseline models in most instances. This visualization enables a clear observation of how each classifier performs across different datasets and highlights OCT-SelfNet's competitive edge in terms of AUC-ROC scores. AUC-ROC is the preferred metric for evaluating classifiers on imbalanced binary datasets because it comprehensively assesses their ability to distinguish between classes across thresholds. This metric focuses on discrimination ability, particularly crucial for imbalanced datasets where one class dominates. Therefore, we chose AUC-ROC to plot our comparison bar chart, ensuring an informative evaluation of classifier performance across various datasets and test sets.

For Tables 2, 3, we performed the Wilcoxon Signed-Rank Test on the AUC-ROC scores of both the baseline model, ResNet50, and our model, OCT-SelfNet-SwinV2. The resulting *p*-value is 0.02, further demonstrating that the difference in AUC-ROC scores is statistically significant.

**Figure 7:**
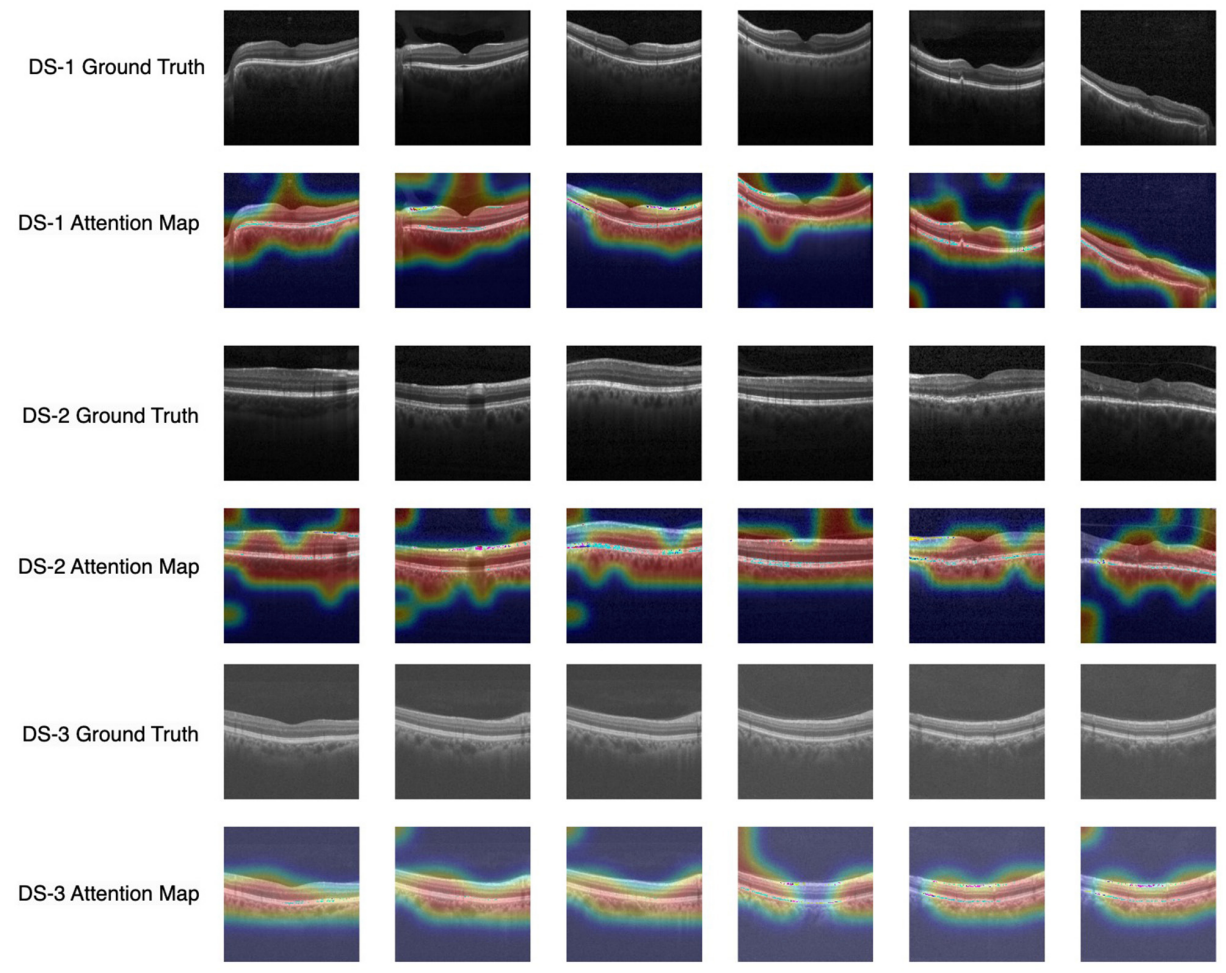
Visualization of model interpretability with attention map overlays highlighting important regions across datasets. Each row corresponds to each dataset, and the subsequent row shows the attention map of the OCT-SelfNet-SwinV2 model overlaid on the ground truth image.

In this performance analysis, the OCT-SelfNet-SwinV2 classifier demonstrates the most reliable performance across all test sets, which is particularly impressive given its smaller size compared to other transformer-based classifiers. Although OCT-SelfNet-Swinlarge slightly outperforms OCT-SelfNet-SwinV2 on Dataset 3, considering both the model's size and its performance scores, SwinV2 proves to be an excellent and efficient option as shown in Table 1. This balance between computational cost and model effectiveness shows the model's potential for deployment in real-world applications where computational efficiency is important. Additionally, a visualization plot in Figure 7 is shown to highlight the OCT-SelfNet-SwinV2 model's decision making process. In this figure, multiple sample images from each dataset is shown, followed by their corresponding attention maps overlaid on top of the original ground truth images. This visualization allows for a better understanding of the regions that were the main focus of the model while making the decision. In the color map, red and yellow areas indicate the most important regions for the model's prediction, receiving the highest attention, while blue areas represent regions with the least attention. From the overlay images, it can be observed that for DS2, which has a comparatively smaller sample size, the attention maps exhibit some noise in the form of additional small highlighted regions which indicates that the model's attention may be less stable due to limited data availability.

**Figure 8:**
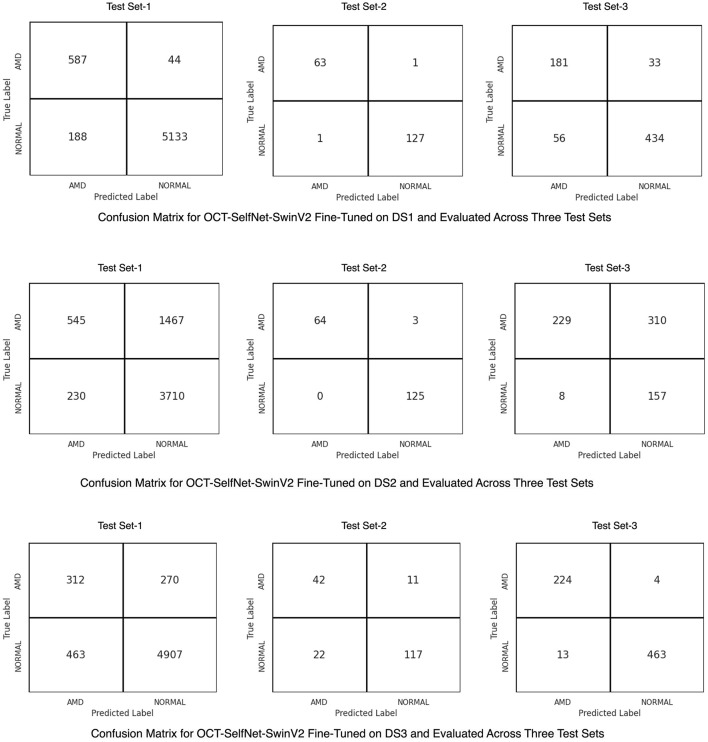
Confusion matrices for the OCT-SelfNet-SwinV2 classifier, trained on DS1 **(Row 1)**, DS2 **(Row 2)**, and DS3 **(Row 3)**. These matrices illustrate the model's performance on its respective test set and generalization across the remaining test sets, providing detailed category-specific insights.

Figure 8 presents the confusion matrix for the OCT-SelfNet-SwinV2 classifier, showcasing its performance after trained on each dataset. In the first row, the matrix illustrates the classifier's effectiveness on Test Set-1 and its generalization across two additional test sets when it was trained on DS1. The second row highlights the results for DS2, while the third row shows the performance for DS3. These confusion matrices provide a comprehensive, category-specific performance analysis, offering deeper insights into the classifier's generalization capabilities across multiple datasets.

**Figure 9:**
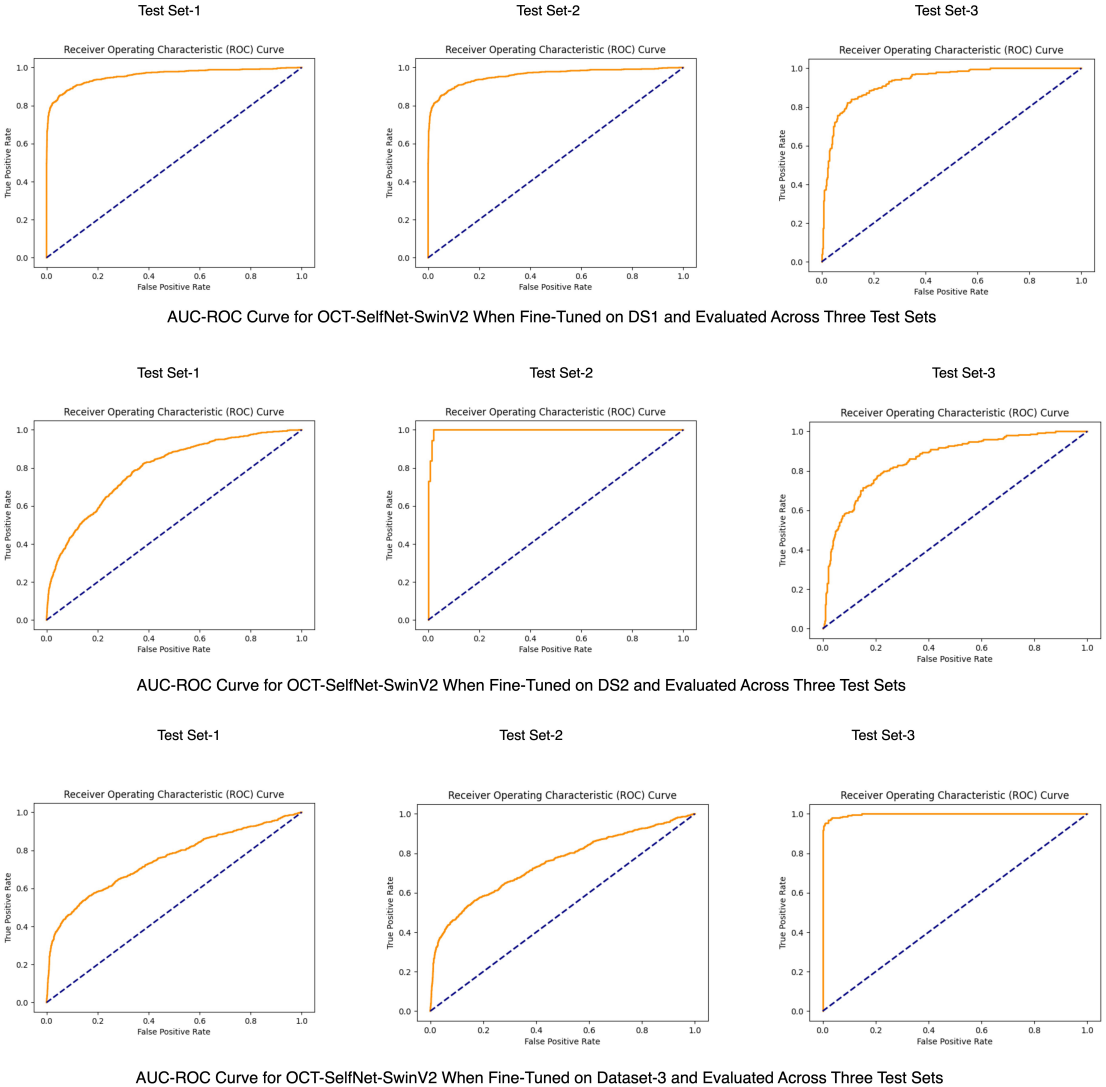
AUC-ROC curves for the OCT-SelfNet-SwinV2 classifier, trained on DS1 **(Row 1)**, DS2 **(Row 2)**, and DS3 **(Row 3)**.

To better visualize the overall performance and the capability of distinguishing categories of OCT-SelfNet-SwinV2 across three datasets, an AUC-ROC curve is shown in Figure 9.

**Figure 10:**
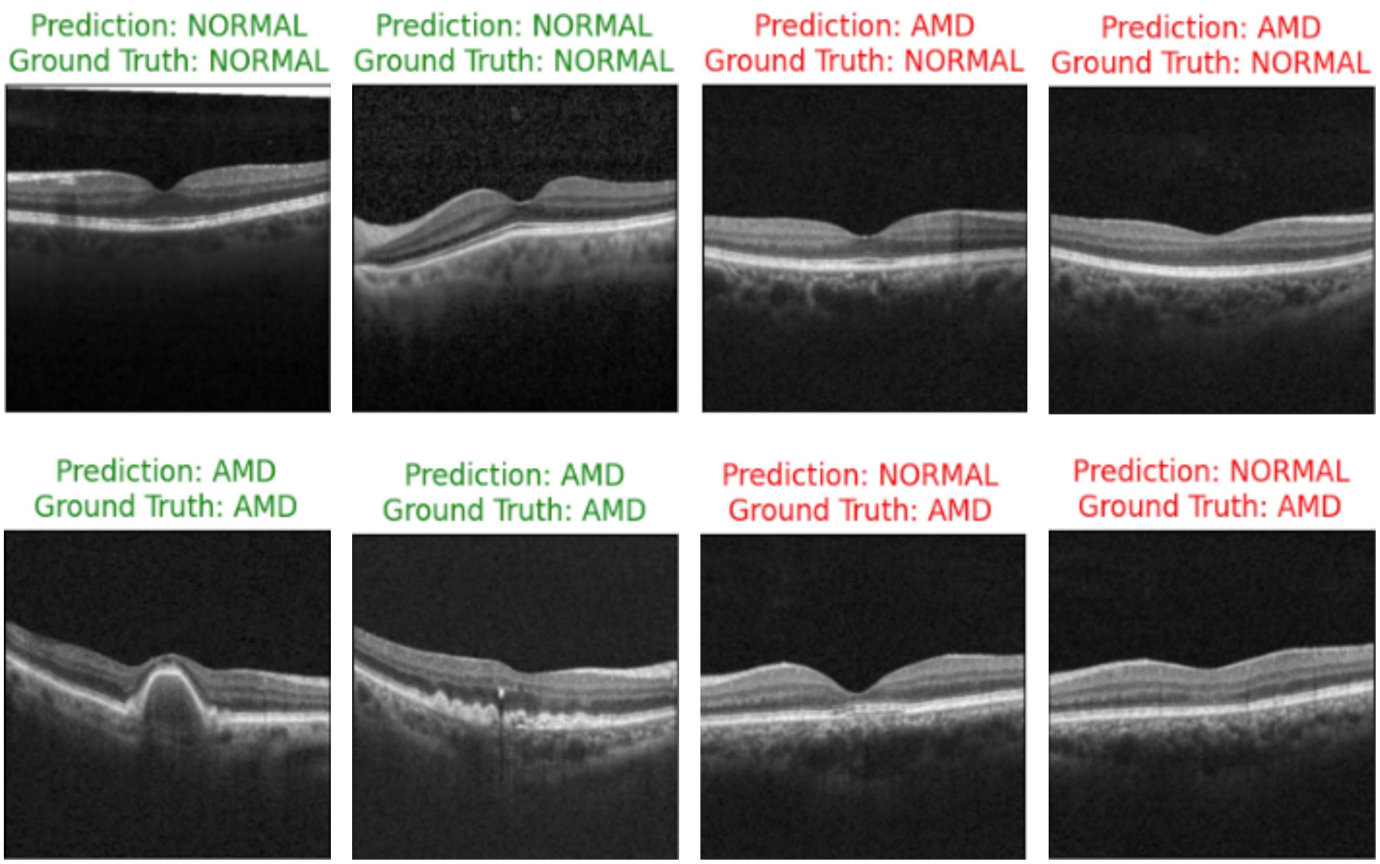
OCT image classification results: this figure displays a series of OCT images with OCT-SelfNet-SwinV2 model predictions and ground truth labels. Each image is annotated with two lines: the first line shows the model's prediction (either AMD or Normal), and the second line indicates the ground truth category. Predictions that match the ground truth are highlighted in green, while incorrect predictions are marked in red.

Figure 10 showcases the results of OCT image classification using the OCT-SelfNet-SwinV2 model. The figure displays a series of OCT images, each annotated with the model's prediction and the corresponding ground truth label. Correct predictions are highlighted in green, and incorrect predictions are marked in red. This visual representation facilitates an intuitive assessment of the model's performance in classifying OCT images across various scenarios, including true positives, true negatives, false positives, and false negatives. For each of these cases, two sample images are provided to illustrate instances where the model succeeded and struggled to make accurate predictions. This analysis reveals that the model encounters difficulties, particularly with visually challenging images to differentiate. In such cases, where the distinctions between categories like AMD and Normal are subtle or ambiguous, the model's predictions are less accurate. This suggests that the model's performance declines when faced with images that require more detailed visual interpretation, highlighting areas where further refinement may be needed.

#### 5.4.2 Performance evaluation on the effect of self-supervised pre-training

This experiment aimed to assess the impact of self-supervised pre-training on overall performance. We conducted experiments where the classifier was trained from scratch without using any pre-trained weights. Our goal was to compare the model's performance with and without self-supervised pre-training. The results, detailed in Table 4 under the names OCT-SelfNet-SwinV2-with-SSL and OCT-SelfNet-SwinV2-without-SSL, show a significant drop in scores for smaller datasets (such as DS2 and DS3) when pre-training was not used. Although not to the same degree, DS1 also showed this drop.

**Table 4:** Analyzing the impact of self-supervised pre-training: comparing our framework with SwinV2 classifier on test sets from three datasets.

**Dataset**	**Classifier name**	**Accuracy**			**AUC-ROC**			**AUC-PR**			**F1-score**		
		**Test1**	**Test2**	**Test3**	**Test1**	**Test2**	**Test3**	**Test1**	**Test2**	**Test3**	**Test1**	**Test2**	**Test3**
DS1	OCT-SelfNet-SwinV2-with-SSL	**0.96**	**0.99**	**0.88**	**0.96**	**0.99**	**0.93**	**0.89**	**0.99**	**0.86**	**0.84**	**0.99**	**0.80**
	OCT-SelfNet-SwinV2-without-SSL	0.95	0.94	0.75	0.95	0.98	0.81	0.87	0.97	0.69	0.79	0.91	0.53
DS2	OCT-SelfNet-SwinV2-with-SSL	0.72	**0.99**	**0.55**	**0.79**	**0.99**	**0.86**	**0.42**	**0.99**	**0.73**	**0.39**	**0.98**	**0.59**
	OCT-SelfNet-SwinV2-without-SSL	**0.73**	0.93	0.44	0.73	**0.99**	0.71	0.24	0.98	0.52	0.36	0.90	0.54
DS3	OCT-SelfNet-SwinV2-with-SSL	**0.88**	0.83	**0.98**	**0.75**	**0.93**	**0.99**	**0.44**	**0.84**	**0.99**	**0.46**	0.72	**0.96**
	OCT-SelfNet-SwinV2-without-SSL	0.84	**0.84**	0.91	0.73	0.88	0.97	0.36	0.81	0.96	0.39	**0.73**	0.87

Evaluation includes accuracy, AUC-ROC, AUC-PR, and F1-score for multi-class classification performance. Scores shown in bold represent the highest performance.

Calculating the *p*-value for the AUC-ROC scores between the OCT-SelfNet-SwinV2-without-SSL and OCT-SelfNet-SwinV2-with-SSL models, we obtained a *p*-value of 0.01, which is significantly low, demonstrating a substantial performance improvement from using the SSL methodology.

These findings suggest that our proposed framework is particularly effective in scenarios where larger datasets are unavailable, as pre-training significantly enhances performance, especially for smaller datasets.

**Figure 11:**
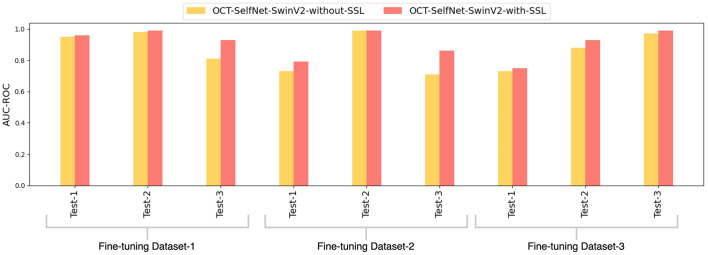
Comparison of AUC-ROC scores for OCT-SelfNet-SwinV2 classifier without and with self-supervised pretraining phase across three datasets and test sets.

Figure 11 displays a bar chart comparing the AUC-ROC scores of the OCT-SelfNet-SwinV2 classifier with and without the self-supervised pre-training stage. The grouped bar chart allows for a side-by-side comparison, clearly indicating that OCT-SelfNet-SwinV2-with-SSL consistently outperforms its counterpart in all evaluated cases.

#### 5.4.3 Performance evaluation on the effect of data fusion in the pre-training phase

In this experiment, we examined the impact of data fusion on classifier performance. Our approach involved using a self-supervised pre-training model based on SwinV2 with individual datasets during training. During the supervised training stage, we used pre-trained weights from the self-supervised model specific to each dataset (DS1, DS2, and DS3). We then compared this approach's performance with a pre-trained model using data fusion. Table 5 presents the performance analysis between the methodologies with and without data fusion for the SwinV2-based network. The results demonstrate a notable decline in performance for smaller datasets (DS2 and DS3) when data fusion was not employed. Conversely, the decline was minimal for DS1, suggesting that data fusion significantly enhances performance, especially for smaller datasets. The *p*-value between the OCT-SelfNet-SwinV2-without-datafusion and OCT-SelfNet-SwinV2-with-datafusion models is 0.01, indicating a significant performance improvement with the use of the data fusion methodology. These findings underscore our proposed methodology's practical relevance and effectiveness in clinical settings.

**Table 5:** Analyzing the impact of data fusion in the pre-training phase: comparing our framework with SwinV2 classifier on test sets from three datasets.

**Dataset**	**Classifier name**	**Accuracy**			**AUC-ROC**			**AUC-PR**			**F1-score**		
		**Test1**	**Test2**	**Test3**	**Test1**	**Test2**	**Test3**	**Test1**	**Test2**	**Test3**	**Test1**	**Test2**	**Test3**
DS1	OCT-SelfNet-SwinV2-with-datafusion	**0.96**	**0.99**	**0.88**	**0.96**	**0.99**	**0.93**	0.89	**0.99**	**0.86**	**0.84**	**0.99**	**0.80**
	OCT-SelfNet-SwinV2-without-datafusion	**0.96**	**0.99**	0.80	**0.96**	**0.99**	0.90	**0.91**	**0.99**	0.81	**0.84**	**0.99**	0.61
DS2	OCT-SelfNet-SwinV2-with-datafusion	**0.72**	**0.99**	**0.55**	**0.79**	**0.99**	**0.86**	**0.42**	**0.99**	**0.73**	**0.39**	**0.98**	**0.59**
	OCT-SelfNet-SwinV2-without-datafusion	0.65	0.90	0.33	0.69	0.97	0.52	0.24	0.95	0.33	0.31	0.86	0.50
DS3	OCT-SelfNet-SwinV2-with-datafusion	**0.88**	**0.83**	**0.98**	**0.75**	**0.93**	**0.99**	**0.44**	**0.84**	**0.99**	**0.46**	**0.72**	**0.96**
	OCT-SelfNet-SwinV2-without-datafusion	0.87	0.80	0.95	0.71	0.88	0.97	0.39	0.80	0.97	0.40	0.65	0.92

Evaluation includes accuracy, AUC-ROC, AUC-PR, and F1-score for multi-class classification performance. Scores shown in bold represent the highest performance.

**Figure 12:**
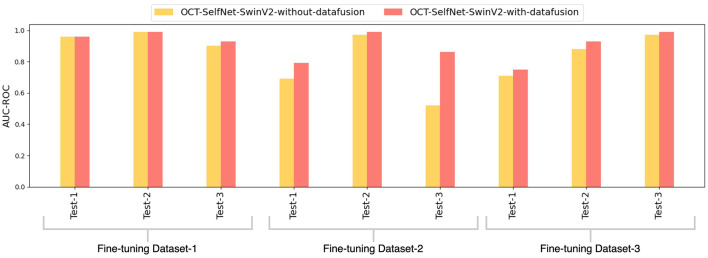
Comparison of AUC-ROC scores for OCT-SelfNet-SwinV2 classifier without and with data fusion methodology across three datasets and test sets.

Figure 12 illustrates a bar chart that contrasts the AUC-ROC scores of the OCT-SelfNet-SwinV2 classifier with and without the integration of the data-fusion methodology. This grouped presentation enables a direct comparison, highlighting the consistent superiority of OCT-SelfNet-SwinV2-with-datafusion across all assessed cases.

#### 5.4.4 Performance evaluation on unseen datasets settings

This experiment aimed to evaluate the performance of our proposed method on datasets that were not part of the pre-training phase; only DS1 was used during pre-training. This allowed us to assess how our method performed on unseen sets, Test Set 2 and Test Set 3, both of which were entirely new to the model which will showcase the practical applicability of our approach in scenarios where there is insufficient data available for training.

**Figure 13:**
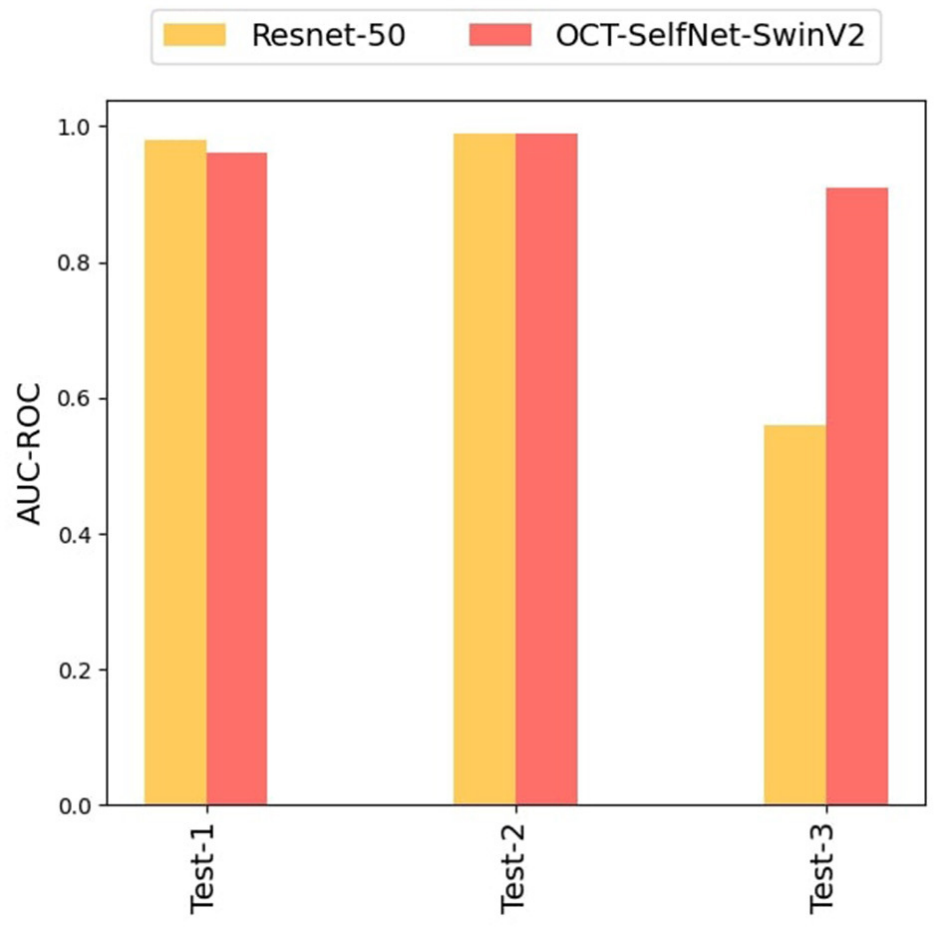
Comparison of OCT-SelfNet-SwinV2 classifier results with baseline model Resnet-50: In OCT-SelfNet-SwinV2, the encoder is pre-trained on only Train Set 1 and later supervised training was done on DS1, evaluated on Test Set 1, Test Set 2, and Test Set 3.

In this experiment, the OCT-SelfNet-SwinV2 underwent pre-training exclusively with DS1, and the acquired weights were subsequently transferred to the classifier network. The classifier network was then trained on DS1, and evaluations were conducted on Test Set 1, Test Set 2, and Test Set 3. For effective pre-training and optimal learning representation, a larger dataset is essential, therefore, DS1 was selected as it has the highest number of samples among the three datasets. The results of this experiment, presented in Table 6, demonstrate that the proposed method consistently outperforms or matches the baseline model across diverse test sets. When evaluating Test Set-1 and Test Set-2, both the baseline model and our approach exhibited similar scores. However, when assessing Test Set-3, which comprises unseen data with variations in image settings, the baseline model's performance significantly declined compared to ours. The narrow score gaps observed in OCT-SelfNet-SwinV2 across diverse test sets highlight the generalization capabilities of our proposed approach. Figure 13 presents a bar chart comparing the AUC-ROC scores for each test set across different classifiers. The grouped bar chart clearly illustrates the superiority of OCT-SelfNet-SwinV2 over the ResNet-50 model in most cases, further confirming its effectiveness in handling a broad spectrum of datasets.

**Table 6:** Comparison of SwinV2-based classifier results, with the encoder pre-trained on Train Set 1 and classifier trained on DS1, followed by evaluation on Test Set 1, Test Set 2, and Test Set 3, in comparison to the baseline model.

**Train set**	**Classifier name**	**Accuracy**			**AUC-ROC**			**AUC-PR**			**F1-score**		
		**Test-1**	**Test-2**	**Test-3**	**Test-1**	**Test-2**	**Test-3**	**Test-1**	**Test-2**	**Test-3**	**Test-1**	**Test-2**	**Test-3**
DS1	Resnet-50	**0.99**	**0.98**	0.70	**0.98**	**0.99**	0.56	**0.97**	0.98	0.70	**0.97**	**0.98**	0.20
	OCT-SelfNet-SwinV2	0.96	0.97	**0.82**	0.96	**0.99**	**0.91**	0.91	**0.99**	**0.84**	0.84	0.96	**0.66**

Scores shown in bold represent the highest performance.

#### 5.4.5 Performance comparison in limited data settings

In this ablation study, the training set was intentionally reduced to 50%, and subsequent training was conducted for each dataset with the augmentation techniques. The objective was to assess the performance of the OCT-SelfNet classifier in comparison to the baseline model under insufficient data conditions.

Examination of Table 7 indicates that, even with a reduction in training data, the proposed method consistently outperforms the baseline model. Specifically, the discrepancy among the scores of unseen test sets compared to the trained dataset's test set is considerable for the baseline model. In contrast, the self-supervised training approach exhibits minimal gaps, showcasing better generalization capabilities. When trained on the reduced DS1 training set, the OCT- SelfNet-SwinV2 model exhibited AUC-ROC scores of 0.96, 0.99, 0.93, and AUC-PR of 0.89, 0.99, 0.83 on Test Set-1, Test Set-2, and Test Set-3, respectively. In comparison, the baseline model achieved AUC-ROC scores of 0.98, 0.99, and 0.55, accompanied by AUC-PR of 0.97, 0.98, and 0.70 on the same test sets. This consistent pattern extended to other datasets, as summarized in Table 7.

**Table 7:** Comparison of our work with the baseline methods (ResNet-50) on test sets from three datasets, using only 50% of the training data in training.

**Train set**	**Classifier name**	**Accuracy**			**AUC-ROC**			**AUC-PR**			**F1-score**		
		**Test-1**	**Test-2**	**Test-3**	**Test-1**	**Test-2**	**Test-3**	**Test-1**	**Test-2**	**Test-3**	**Test-1**	**Test-2**	**Test-3**
DS1	Resnet50	**0.99**	**0.99**	0.70	**0.98**	**0.99**	0.55	**0.97**	0.98	0.70	**0.97**	**0.98**	0.17
	OCT-SelfNet-ViT	0.94	0.94	0.76	0.94	0.98	0.85	0.84	0.97	0.79	0.74	0.91	0.46
	OCT-SelfNet-Swinlarge	0.95	0.97	**0.78**	0.94	**0.99**	0.86	0.85	**0.99**	0.75	0.77	0.96	**0.61**
	OCT-SelfNet-SwinV2	0.96	0.97	**0.78**	0.96	**0.99**	**0.93**	0.89	**0.99**	**0.83**	0.82	0.95	0.54
DS2	Resnet50	**0.79**	0.85	0.33	0.56	0.78	0.50	0.28	0.85	0.66	0.23	0.71	0.50
	OCT-SelfNet-ViT	0.70	0.87	**0.49**	0.73	0.96	**0.83**	0.28	0.94	**0.67**	0.35	0.83	**0.57**
	OCT-SelfNet-Swinlarge	0.68	0.93	0.47	0.70	**0.99**	0.71	0.26	0.96	0.55	0.32	**0.91**	0.55
	OCT-SelfNet-SwinV2	0.72	**0.94**	0.34	**0.76**	**0.99**	0.63	**0.31**	**0.98**	0.41	**0.37**	**0.91**	0.50
DS3	Resnet50	0.77	**0.87**	**0.98**	**0.75**	0.89	**0.99**	**0.54**	0.85	**0.98**	**0.45**	**0.83**	**0.98**
	OCT-SelfNet-ViT	**0.88**	0.79	0.95	**0.75**	0.92	**0.99**	0.41	0.86	**0.98**	0.32	0.58	0.92
	OCT-SelfNet-Swinlarge	**0.88**	0.78	0.95	0.72	0.87	**0.99**	0.41	0.79	**0.98**	0.28	0.55	0.92
	OCT-SelfNet-SwinV2	**0.88**	0.79	0.95	0.64	**0.96**	0.98	0.33	**0.93**	**0.98**	0.29	0.55	0.92

The evaluation focuses on binary classification accuracy, AUC-ROC, AUC-PR, and F1-score. Scores shown in bold represent the highest performance.

**Figure 14:**
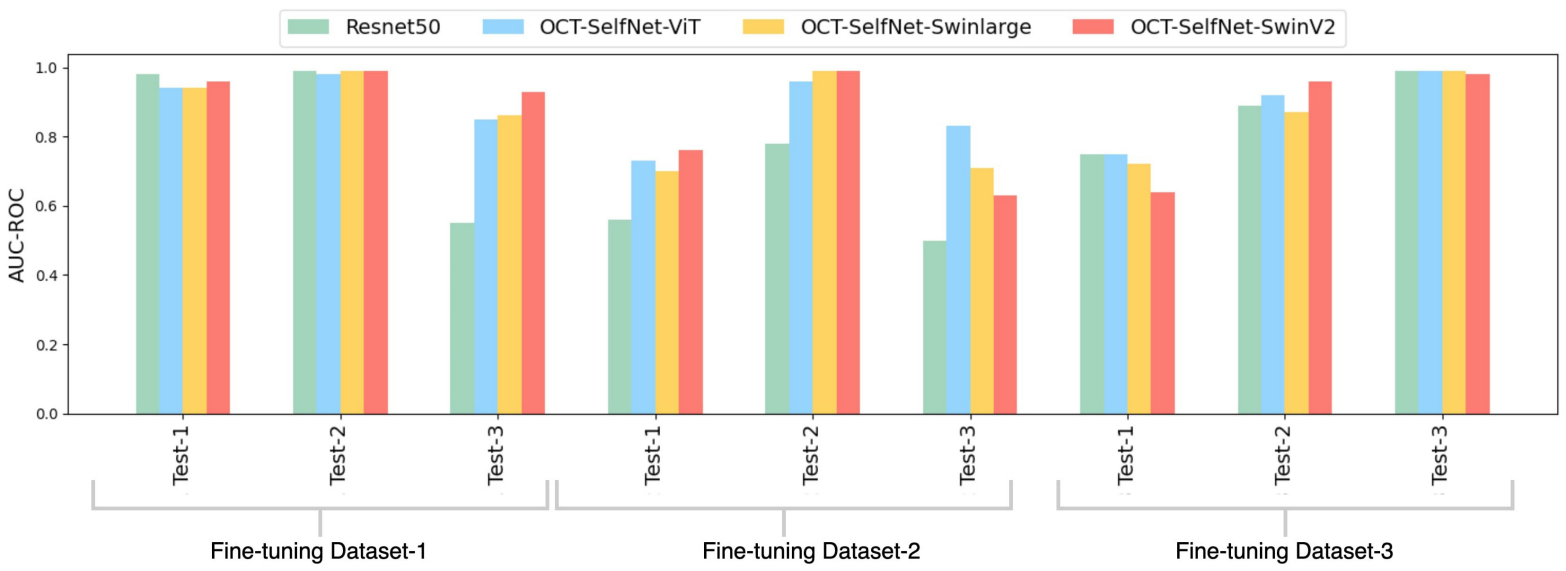
Comparison of AUC-ROC scores between our method (OCT-SelfNet-SwinV2) and baseline (ResNet-50) on test sets from three datasets, when only 50% of training data has been used for training.

In the bar chart shown in Figure 14, AUC-ROC scores for each test set are compared across different classifiers for this experiment, clearly demonstrating how our proposed methodology surpasses the baseline performances.

This finding underscores the robustness and improved adaptability of the proposed methodology, particularly in scenarios with limited training data.

### 5.5 Evaluating the influence of reconstruction quality on the classifier's performance

In this ablation study, a side-by-side comparison was conducted to visually illustrate whether the reconstruction quality during the SSL training phase affects the performance of the trained classifier. By comparing the images from both phases, we aim to observe the relationship between the fidelity of reconstructed images and the subsequent accuracy of classifications made by the model after training.

**Figure 15:**
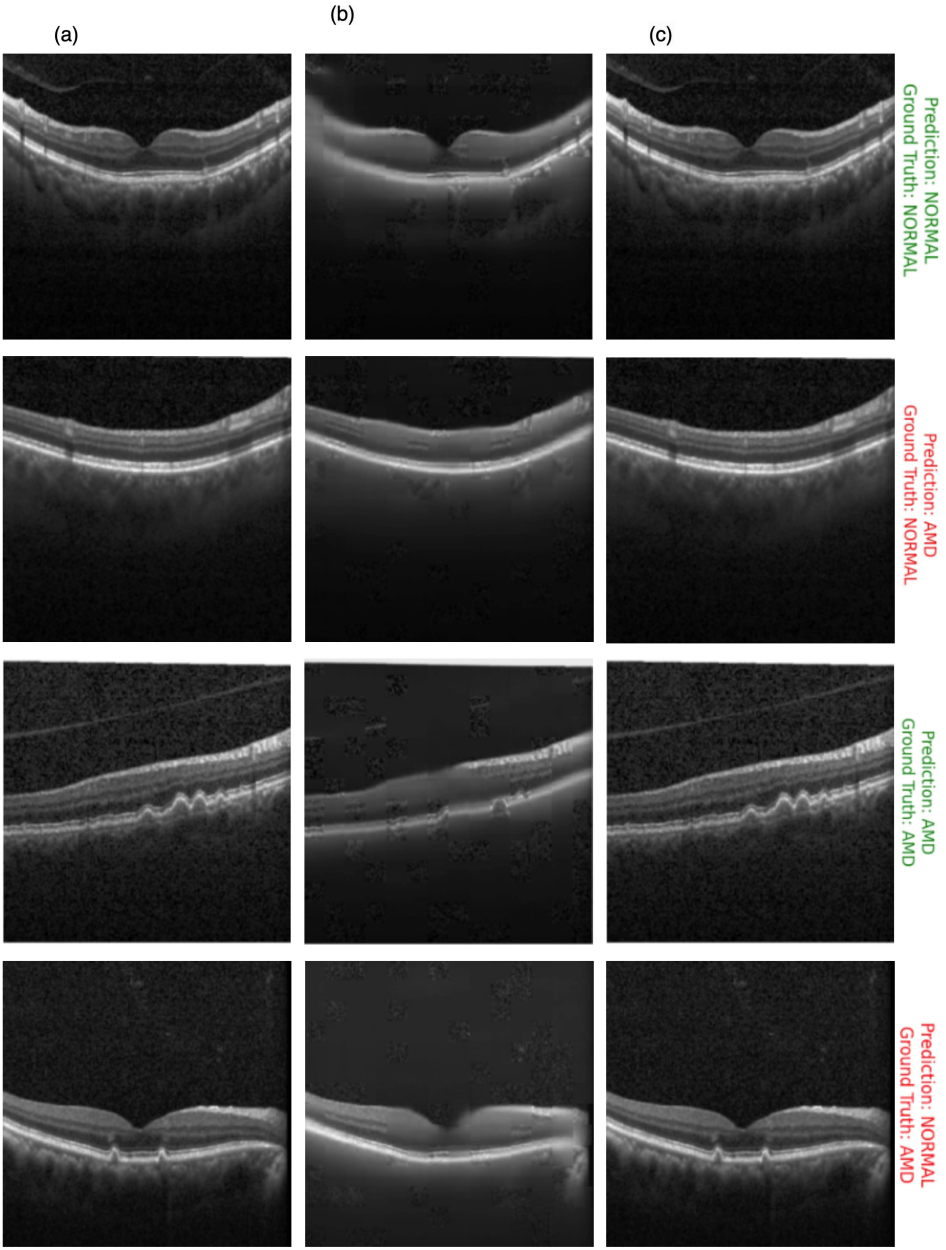
Comparative analysis of **(a**) ground truth, **(b)** reconstructed, and **(c)** classified images by the OCT-SelfNet-SwinV2 model, illustrating the effect of the SSL's performance in accurately classifying the image.

Figure 15 presents a comparative series of images for visual analysis. Figure 15a represents the ground truth image. Figure 15b, depicts the reconstructed output generated by the OCT-SelfNet-SwinV2 model in the self-supervised pre-training phase, showcasing its capability to approximate the original image. Figure 15c illustrates the classification result produced by the OCT-SelfNet-SwinV2 Classifier. The prediction labels are color-coded: green indicates the right prediction while red indicates a wrong prediction. To comprehensively evaluate the impact of reconstruction quality on classification, samples are provided for true-positive, true-negative, false-positive, and false-negative cases. This approach allows us to observe and analyze whether the quality of the reconstructed images influences the accuracy of the classification results. Through this qualitative analysis, it is evident that during the SSL phase, the model adeptly learns the image representation. Despite some minor blurriness in the reconstructed image, these slight imperfections have no impact on the supervised stage. During training, the classifier model effectively uses the weights obtained from the SSL stage to achieve accurate classifications.

## 6 Summary

In this work, we propose a two-phase deep learning framework for detecting eye diseases from OCT images using a SwinV2 backbone. The methodology includes self-supervised pre-training on unlabeled data, followed by supervised training on labeled data. The model is pre-trained using unlabeled OCT images to learn feature representations, addressing data scarcity in medical domains. Using multi-source datasets exposes the model to diverse variations, enhancing generalization. In the training phase, the pre-trained model is adapted for disease classification using labeled OCT images. This phase improves performance while leveraging the learned features, minimizing the need for large labeled datasets. The two-phase approach allows us to address the key limitations in medical AI, such as data scarcity and the need for models that can generalize across diverse clinical environments. Self-supervised learning reduces the dependency on large labeled datasets, which are difficult to obtain in medical imaging. The use of multi-source datasets ensures that the model is exposed to variations in patient demographics, OCT devices, and imaging protocols, thus enhancing its scalability and adaptability for real-world clinical applications.

Our experiments, conducted across three diverse datasets with cross-evaluation and an extensive ablation study with different encoder networks, yield promising results. The performance analysis of the self-supervised training approach against the baseline model reveals significant improvements across various evaluation metrics, notably AUC-ROC and AUC-PR. The consistent outperformance of the SwinV2-based classifier underscores its robustness and suitability for automated ophthalmic diagnosis, specifically in distinguishing normal cases from AMD using OCT images. In our study, we conducted an ablation analysis to assess the impact of self-supervised pre-training on model performance, highlighting its crucial role. It shows a significant decrease in scores for smaller datasets without self-supervised pre-training, emphasizing the framework's effectiveness in enhancing generalization and prediction accuracy, even with smaller datasets. This finding is especially relevant in clinical settings where extensive labeled data acquisition isn't always feasible. Another ablation study was conducted to assess performance under limited data conditions by reducing the sample size to 50%, which further corroborates the robustness and improved adaptability of our methodology for smaller datasets. Despite a reduction in training data, our self-supervised training approach showcases its ability to generalize effectively and maintain performance stability across diverse test sets. The ablation study of unseen datasets settings, where only DS1 was used in the pre-training and test sets from DS2 and DS3 were used for the evaluation, further validates the proposed method's efficacy and generalization abilities for new datasets, showcasing its practical applicability and resilience in handling diverse and complex data in real-world clinical contexts.

Our study acknowledges the potential limitations of the proposed framework that may impact its generalizability and applicability during clinical deployment. One key challenge is the possibility of unwanted biases arising during data fusion, such as imbalances in demographic representation or the prevalence of specific disease categories, which can limit the model's performance. In clinical settings, the model will need to be retrained or trained periodically as new data becomes available. Furthermore, given the high stakes of medical diagnoses, a human-in-the-loop validation process is necessary for low-confidence results to ensure the reliability of the predictions. Our future directions include expanding the dataset to encompass a broader range of patients while addressing and mitigating biases. Additionally, the focus of our current work on binary classification (Normal vs. AMD) limits the scope of the model's practical utility, as real-world clinical settings often involve patients with multiple conditions. We aim to extend the framework to multi-class classification and incorporate human-in-the-loop, making it suitable and reliable for widespread application in ophthalmic diagnostics.

## 7 Conclusion

In this article, we have demonstrated the effectiveness of a self-supervised deep learning framework using the SwinV2 backbone for detecting eye diseases from OCT images. While the shifted window-based transformer model has gained attention in research, its application and self-supervised methodology in OCT analysis have yet to be explored. This article presents a comprehensive two-phase framework that learns feature representations during the pre-training stage from a multi-source dataset and leverages these learned weights for the downstream classification task. Our method reduces the need for large labeled datasets by utilizing unlabeled data in the self-supervised pre-training stage. This approach not only minimizes the dependency on a single data source but also enables the model to learn from the variations across different data settings from multiple sources, improving its generalization capability.

The performance evaluation of our proposed methodology, OCT-SelfNet-SwinV2, through several experiments and scenarios consistently demonstrates its superiority over the baseline model in terms of generalization, showcasing its potential for broader clinical AI applications in automated ophthalmic diagnosis. Our findings highlight the critical importance of self-supervised pre-training, and data fusion in achieving better performance and generalization capabilities. However, our study recognizes potential limitations, including biases from data fusion and the binary classification focus, which may affect generalizability. Future work will address these challenges by expanding the dataset, incorporating multi-class classification, and integrating human-in-the-loop validation for enhanced reliability in clinical applications.

## Data Availability

Publicly available datasets were analyzed in this study. This data can be found at: 1. https://data.mendeley.com/datasets/rscbjbr9sj/2?__hstc=25856994.50884f215aa05251fa08928315486661.1744243773329.1744243773329.1744243773329.1&__hssc=25856994.1.1744243773329&__hsfp=1174180583; 2. https://people.duke.edu/~sf59/Srinivasan_BOE_2014_dataset.htm; 3. https://ieee-dataport.org/open-access/octa-500.

## References

[B1] Alam M. Zhang Y. Lim J. I. Chan R. Yang M. Yao X.. (2020). Quantitative OCT angiography features for objective classification and staging of diabetic retinopathy. Retina 40, 322–332. 10.1097/IAE.0000000000002373 31972803 PMC6494740

[B2] Alshammari H. Gasmi K. Ben Ltaifa I. Krichen M. Ben Ammar L. Mahmood M. A.. (2022). Olive disease classification based on vision transformer and CNN models. Comput. Intell. Neurosci. 2022:3998193. 10.1155/2022/3998193 35958771 PMC9357740

[B3] Awais M. Müller H. Tang T. B. Meriaudeau F. (2017). “Classification OF SD-OCT images using a deep learning approach,” in 2017 IEEE International Conference on Signal and Image Processing Applications (ICSIPA) (Kuching: IEEE), 489–492. 10.1109/ICSIPA.2017.8120661

[B4] Ayana G. Dese K. Dereje Y. Kebede Y. Barki H. Amdissa D.. (2023). Vision-transformer-based transfer learning for mammogram classification. Diagnostics 13:178. 10.3390/diagnostics13020178 36672988 PMC9857963

[B5] Bao H. Dong L. Piao S. Wei F. (2021). Beit: Bert pre-training of image transformers. arXiv [Preprint]. arXiv:2106.08254. 10.48550/arXiv.2106.0825439164302

[B6] Devlin J. Chang M.-W. Lee K. Toutanova K. (2018). Bert: pre-training of deep bidirectional transformers for language understanding. arXiv [Preprint]. arXiv:1810.04805. 10.48550/arXiv:1810.04805

[B7] Dosovitskiy A. Beyer L. Kolesnikov A. Weissenborn D. Zhai X. Unterthiner T.. (2020). An image is worth 16x16 words: transformers for image recognition at scale. arXiv [Preprint]. arXiv:2010.11929. 10.48550/arXiv.2010.11929

[B8] Fang L. Guo J. He X. Li M. (2022). Self-supervised patient-specific features learning for OCT image classification. Med. Biol. Eng. Comput. 60, 2851–2863. 10.1007/s11517-022-02627-8 35931872

[B9] Friberg T. R. Bilonick R. A. Brennen P. M. (2011). Analysis of the relationship between drusen size and drusen area in eyes with age-related macular degeneration. Ophthalmic Surg. Lasers Imaging Retina 42, 369–375. 10.3928/15428877-20110812-01 21899243

[B10] Gholami S. Lim J. I. Leng T. Ong S. S. Y. Thompson A. C. Alam M. N.. (2023). Federated learning for diagnosis of age-related macular degeneration. Front. Med. 10:1259017. 10.3389/fmed.2023.1259017 37901412 PMC10613107

[B11] He K. Chen X. Xie S. Li Y. Dollár P. Girshick R. (2022). “Masked autoencoders are scalable vision learners,” in Proceedings of the IEEE/CVF Conference on Computer vision and Pattern Recognition (New Orleans, LA: IEEE), 16000–16009. 10.1109/CVPR52688.2022.01553

[B12] He K. Zhang X. Ren S. Sun J. (2015). Deep residual learning for image recognition. arXiv [Preprint]. arXiv:1512.03385. 10.48550/arXiv.1512.03385

[B13] He Y. Zhu D. Chen H. Fan T. Xiao X. Cai Y.. (2023). Longitudinal deep network for consistent oct layer segmentation. Biomed. Optics Express 14, 1874–1893. 10.1364/BOE.487518 37206119 PMC10191669

[B14] Jing L. Tian Y. (2020). Self-supervised visual feature learning with deep neural networks: a survey. IEEE Trans. Pattern Anal. Mach. Intell. 43, 4037–4058. 10.1109/TPAMI.2020.2992393 32386141

[B15] Kermany D. S. Goldbaum M. Cai W. Valentim C. C. Liang H. Baxter S. L.. (2018). Identifying medical diagnoses and treatable diseases by image-based deep learning. Cell 172, 1122–1131. 10.1016/j.cell.2018.02.010 29474911

[B16] Kihara Y. Shen M. Shi Y. Jiang X. Wang L. Laiginhas R.. (2022). Detection of nonexudative macular neovascularization on structural OCT images using vision transformers. Ophthalmol. Sci. 2:100197. 10.1016/j.xops.2022.100197 36531577 PMC9754966

[B17] Leandro I. Lorenzo B. Aleksandar M. Rosa G. Agostino A. Daniele T.. (2023). Oct-based deep-learning models for the identification of retinal key signs. Sci. Rep. 13:14628. 10.1038/s41598-023-41362-4 37670066 PMC10480174

[B18] Lee C. Baughman D. Lee A. (2017). Deep learning is effective for the classification of OCT images of normal versus age-related macular degeneration. Ophthalmol. Retina 1, 322–327. 10.1016/j.oret.2016.12.009 30693348 PMC6347658

[B19] Leingang O. Riedl S. Mai J. Reiter G. S. Faustmann G. Fuchs P.. (2023). Automated deep learning-based AMD detection and staging in real-world OCT datasets (pinnacle study report 5). Sci. Rep. 13:19545. 10.1038/s41598-023-46626-7 37945665 PMC10636170

[B20] Li L. Zhang T. Zhang X. Liu J. Ma B. Luo Y.. (2024). Medflip: medical vision-and-language self-supervised fast pre-training with masked autoencoder. arXiv preprint arXiv:2403.04626.

[B21] Li M. Huang K. Xu Q. Yang J. Zhang Y. Ji Z.. (2020). Octa-500: a retinal dataset for optical coherence tomography angiography study. arXiv [Preprint] arXiv:2012.07261. 10.48550/arXiv.2012.07261 38325155

[B22] Liu Z. Hu H. Lin Y. Yao Z. Xie Z. Wei Y.. (2022). “Swin transformer v2: scaling up capacity and resolution,” in Proceedings of the IEEE/CVF Conference on Computer Vision and Pattern Recognition (New Orleans, LA: IEEE), 12009–12019. 10.1109/CVPR52688.2022.01170

[B23] Liu Z. Lin Y. Cao Y. Hu H. Wei Y. Zhang Z.. (2021). “Swin transformer: Hierarchical vision transformer using shifted windows,” in Proceedings of the IEEE/CVF International Conference on Computer Vision (Montreal, QC: IEEE), 10012–10022. 10.1109/ICCV48922.2021.00986

[B24] Loshchilov I. Hutter F. (2017). Decoupled weight decay regularization. arXiv preprint arXiv:1711.05101.38536692

[B25] Lu W. Tong Y. Yu Y. Xing Y. Chen C. Shen Y.. (2018). Deep learning-based automated classification of multi-categorical abnormalities from optical coherence tomography images. Transl. Vis. Sci. Technol. 7, 41–41. 10.1167/tvst.7.6.41 30619661 PMC6314222

[B26] Mukherjee S. Girard T. Welfer D. Seabra J. C. Aubert V. Vallès-Saiz L.. (2022a). Retinal layer segmentation in optical coherence tomography (OCT) using a 3D deep-convolutional regression network for patients with age-related macular degeneration. Biomed. Optics Express 13, 3195–3210. 10.1364/BOE.450193 35781941 PMC9208604

[B27] Mukherjee S. Silva T. D. Jayakar G. Grisso P. Wiley H. Keenan T.. (2022b). “Retinal layer segmentation for age-related macular degeneration patients with 3D-UNet,” in Medical Imaging 2022: Computer-Aided Diagnosis, Volume 12033, eds. K. Drukker, and K. M. Iftekharuddin (San Diego, CA: International Society for Optics and Photonics, SPIE), 12033J. 10.1117/12.2612991

[B28] Okolo G. I. Katsigiannis S. Ramzan N. (2022). Ievit: an enhanced vision transformer architecture for chest X-ray image classification. Comput. Methods Programs Biomed. 226:107141. 10.1016/j.cmpb.2022.107141 36162246

[B29] Qiu J. Sun Y. (2019). Self-supervised iterative refinement learning for macular OCT volumetric data classification. Comput. Biol. Med. 111:103327. 10.1016/j.compbiomed.2019.103327 31302456

[B30] Schmidt-Erfurth U. Waldstein S. M. Klimscha S. Sadeghipour A. Hu X. Gerendas B. S.. (2018). Prediction of individual disease conversion in early amd using artificial intelligence. Invest. Ophthalmol. Vis. Sci. 59, 3199–3208. 10.1167/iovs.18-24106 29971444

[B31] Scott A. W. Bressler S. B. (2013). Long-term follow-up of vascular endothelial growth factor inhibitor therapy for neovascular age-related macular degeneration. Curr. Opin. Ophthalmol. 24, 190–196. 10.1097/ICU.0b013e32835fefee 23492430

[B32] Sotoudeh-Paima S. Jodeiri A. Hajizadeh F. Soltanian-Zadeh H. (2022). Multi-scale convolutional neural network for automated AMD classification using retinal OCT images. *Comput*. Biol. Med. 144:105368. 10.1016/j.compbiomed.2022.105368 35259614

[B33] Srinivasan P. P. Kim L. A. Mettu P. S. Cousins S. W. Comer G. M. Izatt J. A.. (2014). Fully automated detection of diabetic macular edema and dry age-related macular degeneration from optical coherence tomography images. Biomed. Optics Express 5, 3568–3577. 10.1364/BOE.5.003568 25360373 PMC4206325

[B34] Tsuji T. Hirose Y. Fujimori K. Hirose T. Oyama A. Saikawa Y.. (2020). Classification of optical coherence tomography images using a capsule network. BMC Ophthalmol. 20, 1–9. 10.1186/s12886-020-01382-4 32192460 PMC7082944

[B35] Vaswani A. Shazeer N. Parmar N. Uszkoreit J. Jones L. Gomez A. N. Kaiser L. Polosukhin I. (2017). Attention is all you need. arXiv preprint arXiv:1706.03762. Available online at: https://arxiv.org/abs/1706.03762

[B36] Wang Q. Xiong Y. Zhu H. Mu X. Zhang Y. Ma Y.. (2024). Cervical OCT image classification using contrastive masked autoencoders with swin transformer. Computerized Med. Imaging Graph. 118:102469. 10.1016/j.compmedimag.2024.102469 39577206

[B37] Wang W. Gawlik K. Lopez J. Wen C. Zhu J. Wu F.. (2016). Genetic and environmental factors strongly influence risk, severity and progression of age-related macular degeneration. Signal Transduct. Target. Ther. 1, 1–6. 10.1038/sigtrans.2016.23 29263899 PMC5661646

[B38] Wang W. Jiang R. Cui N. Li Q. Yuan F. Xiao Z.. (2022). Semi-supervised vision transformer with adaptive token sampling for breast cancer classification. Front. Pharmacol. 13:929755. 10.3389/fphar.2022.929755 35935827 PMC9353650

[B39] Wolf D. Payer T. Lisson C. S. Lisson C. G. Beer M. Götz M.. (2023). Self-supervised pre-training with contrastive and masked autoencoder methods for dealing with small datasets in deep learning for medical imaging. Sci. Rep. 13:20260. 10.1038/s41598-023-46433-0 37985685 PMC10662445

[B40] World Health Organization (2023). Blindness and Visual Impairment. Geneva: WHO.

[B41] Xu W. Fu Y.-L. Zhu D. (2023). Resnet and its application to medical image processing: research progress and challenges. Comput. Methods Programs Biomed. 240:107660. 10.1016/j.cmpb.2023.107660 37320940

[B42] Yi K. Mujat M. Park B. H. Sun W. Miller J. W. Seddon J. M. T. (2009). Spectral domain optical coherence tomography for quantitative evaluation of drusen and associated structural changes in non-neovascular age-related macular degeneration. Br. J. Ophthalmol., 93, 176–181. 10.1136/bjo.2008.137356 18697811 PMC2628537

[B43] Zhou L. Liu H. Bae J. He J. Samaras D. Prasanna P.. (2023). “Self pre-training with masked autoencoders for medical image classification and segmentation,” in 2023 IEEE 20th International Symposium on Biomedical Imaging (ISBI). (Cartagena: IEEE). 10.1109/ISBI53787.2023.10230477

